# Super-enhancers in tumors: unraveling recent advances in their role in Oncogenesis and the emergence of targeted therapies

**DOI:** 10.1186/s12967-025-06098-x

**Published:** 2025-01-21

**Authors:** Yumeng Ji, Baixue Li, Rongjin Lin, Jing Yuan, Yang Han, Yuping Du, Yang Zhao

**Affiliations:** 1 Department of Obstetrics and Gynecology, Department of Gynecologic Oncology Research Office, Guangzhou Key Laboratory of Targeted Therapy for Gynecologic Oncology, Guangdong Provincial Key Laboratory of Major Obstetric Diseases, Guangdong Provincial Clinical Research Center for Obstetrics and Gynecology, Guangdong-Hong Kong-Macao Greater Bay Area Higher Education Joint Laboratory of Maternal-Fetal Medicine, The Third Affiliated Hospital, Guangzhou Medical University, Guangzhou, China; 2 No.63 Duobao Road, Liwan District, Guangzhou City, Guangdong Province P.R. China

**Keywords:** Super enhancer, Transcription regulatory, Cancer, Targeted therapy

## Abstract

Super enhancers are a unique class of enhancers that possess a distinct structure and mechanism, which enable them to exhibit stronger gene transcription regulatory function than classical enhancers, thereby regulating cellular activities. In tumor samples, super enhancers have been identified as crucial players in the development and progression of tumor cells, opening up new avenues for cancer research and treatment. This review provides a concise overview of various models regarding super enhancer assembly and activation, examining the mechanisms through which tumor cells acquire or activate these enhancers and regulate carcinogenic transcription programs. Furthermore, we discuss the current landscape and challenges in developing cancer therapeutic drugs that target super enhancers.

## Introduction

Transcriptional dysregulation is a key mechanism underlying tumor development, involving changes in protein-coding genes or non-coding regulatory elements. In this article, we will discuss a unique class of enhancers called super enhancers (SE) that play a crucial role as transcriptional regulatory elements in maintaining the tumorigenic properties of cancer cells. The conventional chemotherapy drugs used in tumor treatment are currently encountering challenges in overcoming tumor resistance. This dilemma has motivated researchers to explore the downregulation of SE function as a therapeutic strategy, aiming to regulate the loss of tumorigenic characteristics in cancer cells and inhibit tumor growth. In this review, we will delve into the formation mechanism, mode of action, and significance of carcinogenic SE in tumors, highlighting the latest research findings related to targeted therapy focused on super enhancers.

To this day, cancer remains one of the most serious public health issues affecting people’s lives and health worldwide. In China and the United States, the incidence rate of lung cancer, breast cancer, prostate cancer and colorectal cancer is increasing year by year [1]. Although a large number of researchers and money are invested every year in the research of cancer mechanisms and the development of new anti-tumor drugs, tumor cell resistance remains the most critical factor hindering the complete cure of cancer. Unfortunately, the emerging small molecule therapies [2] and immunotherapy [3] have not achieved their ideal treatment goals due to the development of tumor cell resistance [4]. We need to consider whether we can start from the mechanism of tumor cell occurrence, search for new targets, and develop related drugs to address the challenges posed by tumor cell resistance.

Whether it is normal cells or tumor cells, the maintenance of any cellular life activity depends on gene expression, and the normal or abnormal expression of genes involves complex mechanisms such as coordinated interactions between trans acting factors, including transcription factors, and cis acting elements, or between cis acting elements themselves, as well as the regulation of transcriptional activation processes by chromatin structure. The characteristics of tumor cell growth and proliferation, including infinite proliferation, immortalization, apoptosis evasion, invasion and metastasis, immune evasion, and drug resistance, are all related to abnormal gene expression [5]. In previous studies, it had been proven that changes in gene sequence or transcriptional dysregulation caused by epigenetic factors was the fundamental mechanism of tumor occurrence and development [6]. Currently, many of the oncogenes and tumor suppressor genes that have been extensively studied belong to genes encoding transcription factors, indicating an important correlation between dysregulation of gene transcription and the occurrence and progression of tumors [5–8]. With the progress of research on the changes in the transcriptional regulation of multiple genes caused by various mutations in tumor cells, we have found that these mutations can have adverse effects on gene regulation, and thus have profound impacts on the tumor genome and epigenome. Therefore, an increasing amount of evidence is pointing out that dysregulation of transcriptional mechanisms plays a central role in the occurrence and development of tumors [7, 8].

Within the realm of transcriptional regulation, the control of the interaction between cis-acting elements and cis-acting elements is an area that has not received much attention. One prominent example of this is the interaction between enhancers and promoters [9]. Enhancers, acting as platforms for transcription factor (TF) binding, are usually 200–500 bp long and consist of several TF recognition binding sites [10]. Their main role is to create a loop structure by attaching to the transcription initiation site, which in turn attracts transcription factors, coactivators, and other mediators to form transcription initiation complexes. This results in the targeted interaction between enhancers and RNA polymerase II in the promoter region in a gene-specific way, ultimately triggering the process of transcription [11]. Crucially, enhancers have the ability to stimulate gene expression without being affected by their distance, position, or direction in relation to the transcription initiation site [12]. Active enhancers exhibit monomethylation at H3 histone lysine 4 (H3K4me1), acetylation at lysine 27 (H3K27ac), and the lack of trimethylation at lysine 4 (H3K4me3). The mixed lineage leukemia (MLL) methyltransferase family (MLL2/3/4) is responsible for catalyzing the alteration of H3K4me1, while the modification of H3K27ac is done by the CREB binding protein (CBP)/p300 acetyltransferase. Concurrently, the elimination of the H3K4me3 marker is aided by the KDM5C demethylase, which specifically targets H3K4me3 [13, 14].

**Fig. 1:**
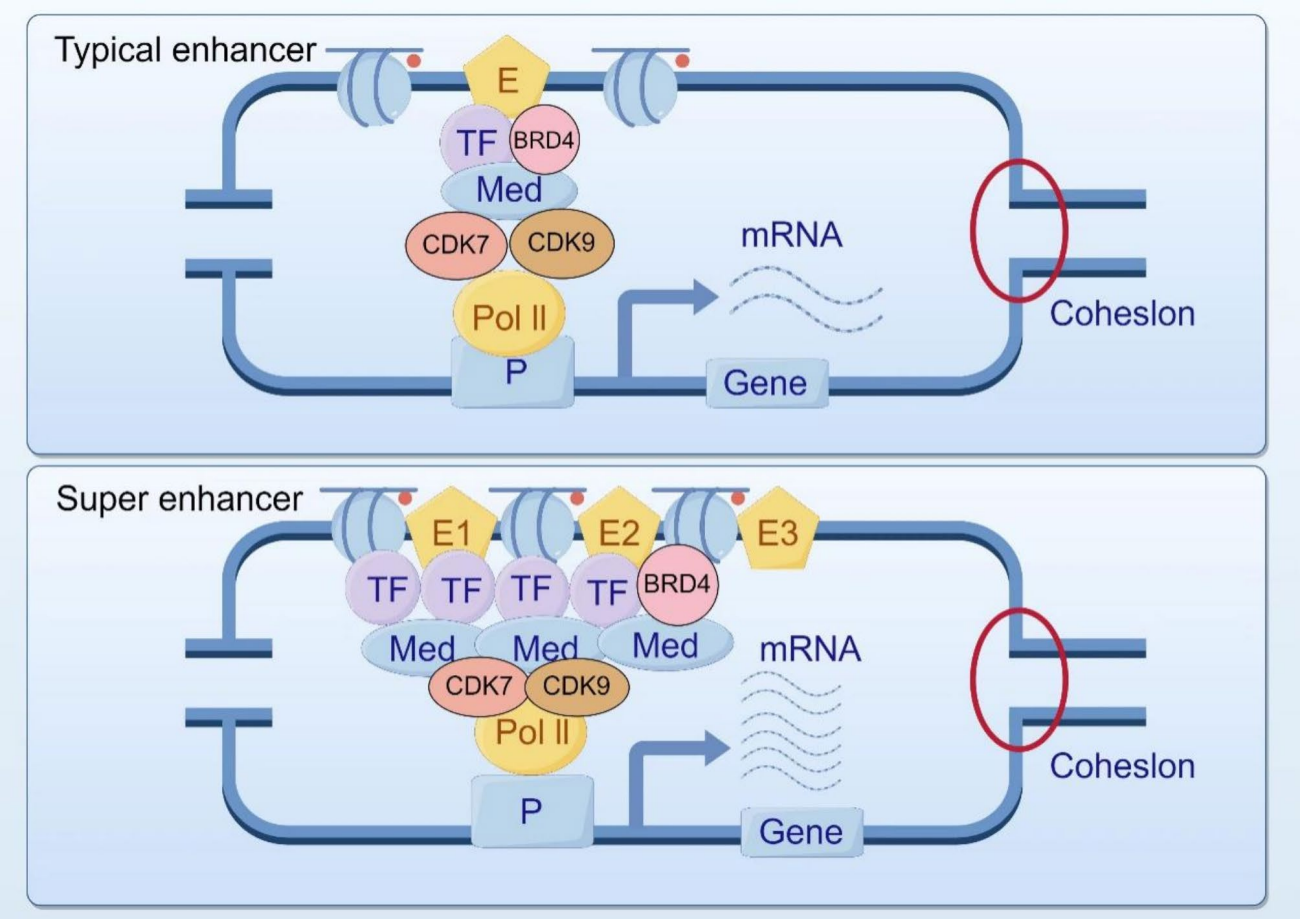
Structural differences between super enhancers and typical enhancers Super enhancers are a set of unique cis-regulatory elements composed of several adjacent enhancers. The unique feature is that the average density of TF and coactivators raised by super enhancers is higher than that of typical enhancers. Therefore, compared with typical enhancers, super enhancers can drive target gene transcription more efficiently (By Figdraw)

Super enhancers (SE) are a unique set of cis regulatory elements comprised of multiple adjacent enhancers, with a total length of approximately 8-20 kb. What distinguishes super enhancers is their higher density of TF and co-activators recruitment, which is on average 10 times higher than traditional classical enhancers. Consequently, super enhancers exhibit a greater efficiency in driving target gene transcription compared to classical enhancers [15–18]. The schematic diagrams of the mechanisms of typical enhancers and super enhancers during their function can be seen in Fig. 1. The first discovery of super enhancers was made by Whyte in mouse embryonic stem cells (ESC), and subsequent studies have identified super enhancers in various cell types [19]. Shortly thereafter, super enhancers were found in tumor cells, and it was discovered that they affect the expression of key oncogenes in various tumor samples. This indicates that super enhancers have a role in regulating oncogene transcription [6, 15, 16, 20]. In recent years, an increasing number of studies have shown countless connections between super enhancers and cancer. In light of the growing number of reports on chemotherapy drug resistance, the exploration of novel tumor treatment methods through super enhancers opens up new avenues for studying tumor mechanisms and treatment. In this review, we provide a brief overview of the current models of super enhancer assembly and activation, discuss different mechanisms by which cancer cells acquire or activate super enhancers and modulate carcinogenic transcription programs, and specifically focus on the development of promising drugs and treatment methods for future cancer treatment using super enhancer targeting.

## The ways of abnormal regulation caused by super - enhancers

Research has found that the super enhancers that promote abnormal expression of oncogenes are mainly formed in the following two ways [21]: [1] dysregulation of transcription regulatory factors; [2] By disrupting the advanced structure of chromatin, including translocation, inversion, amplification and insertion, deletion, etc., super enhancers are unexpectedly formed near oncogenes. The latest research suggests that there may be a third way of formation, which is the mutation of the super enhancer itself.

### The dysregulation of transcription regulatory factors

The imbalance of transcription regulatory factors mainly includes three situations [21]: [1] The production of chimeric transcription factors; [2] Changes in the expression, function, and stability of transcription regulatory factors; [3] Cross talk between transcription regulatory factors. These three situations have been reported in multiple tumor related literature.

#### Production of chimeric transcription factors

Hematological cancers frequently include the development of chimeric transcription factors (TF). An example of this is the TCF3-HLF chimeric transcription factor (TF) seen in juvenile acute lymphoblastic leukemia (ALL). It specifically attaches to HLF binding sites located in super enhancers associated with hematopoietic stem cells and bone marrow lineage. This binding then triggers a transformation program mediated by the MYC gene, which is conserved across different organisms. Nevertheless, when p300 is inhibited, the TCF3-HLF enhancement subroutine loses its effectiveness and has a significant impact in preventing leukemia [22].

In childhood acute megakaryocytic leukemia (AMKL), the ETO2-GLIS2 chimeric transcription factor (TF) builds up in the super enhancer of leukemia cells [23]. The binding region of ETO2-GLIS2 contains a high concentration of the DNA binding motif of GLIS2 and multiple recognized partners of ETO2, such as ERG (ETS), GATA, and RUNX. Significantly, more than 50% of the binding sites in normal cells do not form connections with ETO2 partners. This suggests that the chimeric transcription factor selectively attaches to distinct novel sites in cancer cells [23].

Recent investigations have emphasized the importance of modified TF properties in hematological malignancies. For instance, the NUP98 fusion chimeric transcription factor in pediatric acute myeloid leukemia (AML) modifies the formation of condensed structures, hence influencing the expression of genes associated with leukemia [24, 25]. In AML with chromosome 16 inversion, CBFβ-SMMHC hinders the activity of RUNX1, the primary transcription factor in the hematological system, and interferes with the suppression of MYC expression, which is regulated by RUNX1 [26, 27]. These findings highlight the significance of comprehending and focusing on chimeric TFs in the advancement of novel treatment approaches for hematological malignancies.

#### Alterations and crosstalk of transcriptional regulators

In the second and third instances, it was found that the improper control of super enhancers in different forms of cancer is linked to alterations in the expression of oncogenic transcription factors, mutations in transcription regulatory factors, and changes in functional crosstalk. Acute myeloid leukemia (AML) is commonly linked to heightened expression of HOXA9, which stimulates the development of leukemia by activating particular enhancers, including super enhancers, in leukemia cells [28]. The protein TRIB1, which is found in bone marrow malignancy, acts as a pseudokinase. It blocks the activity of C/EBPα P42 and controls the super enhancer that binds to HOXA9. This process speeds up the development of leukemia caused by HOXA9 [29].

The NUP214-ABL1 fusion kinase, which is frequently observed in acute lymphoblastic leukemia (ALL), has a vital function in facilitating the cooperative attachment of TLX1 and STAT5 to enhancers, thus triggering the activation of significant oncogenes such as MYC and BCL2 [30]. Super enhancers, commonly found in follicular lymphoma (FL) and diffuse large B-cell lymphoma (DLBCL), often acquire functional loss mutations in the CREBBP acetyltransferase and its analogous counterpart p300 [31]. The mutation in CREBBP is thought to interfere with the activity of the transcriptional suppressor BCL6 and hinder genetic control in the germinal center, where it is governed by a super enhancer [31]. In addition, FL (follicular lymphoma) and DLBCL (diffuse large B-cell lymphoma) frequently have mutations in MEF2B, a transcription factor that is highly concentrated in germinal center-specific super enhancers [32]. It is believed that these MEF2B mutations interfere with gene expression by reducing the ability to bind to DNA and destabilizing the protein. MEF2B effectively avoids negative regulation by forming HUCA complexes [32].

Furthermore, cancer caused by viruses is also linked to modifications in super enhancers. Primary exudative lymphoma (PEL) is characterized by the interaction between Kaposi’s sarcoma-associated herpesvirus (KSHV)-driven transcription factors and viral interferon regulatory factor 3 (vIRF3) with host transcription factors. This partnership facilitates the activation of survival genes through the involvement of super enhancers, such as IRF4 and BATF [33, 34]. These findings highlight the importance of understanding the complex interaction between cancer-causing transcription factors, genetic alterations, and functional communication in the control of super enhancers throughout the genesis of cancer.

### Disruption of advanced chromatin structure

Chromatin alterations like as translocations, inversions, amplifications, insertions, deletions, and other abnormalities can lead to the formation of new super enhancers in the vicinity of oncogenes. The presence of anomalies in well-established oncogenes like as MYC and N-MYC can result in the creation of abnormal super enhancers and the disruption of oncogene expression in various types of cancer [15]. In cases of malignant lymphoma, chromosomal translocations have the ability to relocate super enhancers to immunoglobulin loci in close proximity to MYC, leading to an elevation in MYC expression. In cases of acute myeloid leukemia (AML), the relocation of the distal GATA2 enhancer due to translocations or inversions of chromosome 3 can result in the abnormal activation of the EVI1 oncogene through the translocation-derived super enhancer. As a result, the GATA2 gene becomes haploid [35, 36].

Over the past few years, instances of “enhancer hijacking” have been detected in solid tumors such medulloblastoma, adenoid cystic carcinoma, and thyroid cancer [37–39]. The PEAR-ChIP method, a high-throughput technique that combines chromatin immunoprecipitation with enhancer activity detection, has been employed to detect and identify different gene rearrangements linked to established cancer types, such as translocations and chromosomal deletions. Several factors implicated in these rearrangements include CCND1, BCL2, MYC, PDCD1LG2, NOTCH1, CIITA, and SGK1. PEAR-ChIP has also discovered previously unknown repetitive enhancer events at the MYC location and enhancers unique to certain subtypes in lymphoma [40]. These findings highlight the significance of comprehending the influence of chromosomal rearrangements on the creation of super enhancers and the disruption of oncogenes in the context of cancer progression.

### Enhancer mutation

Enhancers have the capability to experience mutations. For instance, in a specific group of T-cell acute lymphoblastic leukemia (T-ALL), there is a brief insertion of nucleotide bases that happens before the TAL1 oncogene [41]. This mutation generates a specific DNA sequence that can bind to the MYB transcription factor. As a result, other transcription factors are recruited, which triggers the creation of super enhancers. This, in turn, activates the TAL1 gene [41]. In recent years, similar evidence has been identified in other tumors as well [42, 43].

A recent in-depth study employed targeted resequencing of cis regulatory elements (CRE) associated with hematopoietic lineage, along with CRISPR/dCas9 gene knockout technology, to detect recurrent mutations in acute myeloid leukemia (AML), lymphoma, and ALL. This analysis identified both carcinogenic and suppressive CRE [44]. The findings of this work demonstrate that mutations in CRE, which are situated in close proximity to the binding sites of nuclear receptors (NR), such as the KRAS and PER2 enhancers, have the potential to impact the response to NR signaling and the expression levels of target genes. In addition, the analysis revealed a prevalent co-localization of CRE and NR binding sites [44]. The comprehension of CRE mutations and their influence on gene regulation and signaling pathways offers vital insights into the molecular mechanisms that underlie different types of malignancies.

## The models of action of super enhancers

In tumor cells, super - enhancers play a role in promoting oncogene transcription. Currently, research has found that the mechanisms mainly fall into two categories. One is the conventional model which has been widely studied, and the other is the phase - separation model proposed by Hnisz D et al. in 2017 [45].

### The conventional model

**Fig. 2:**
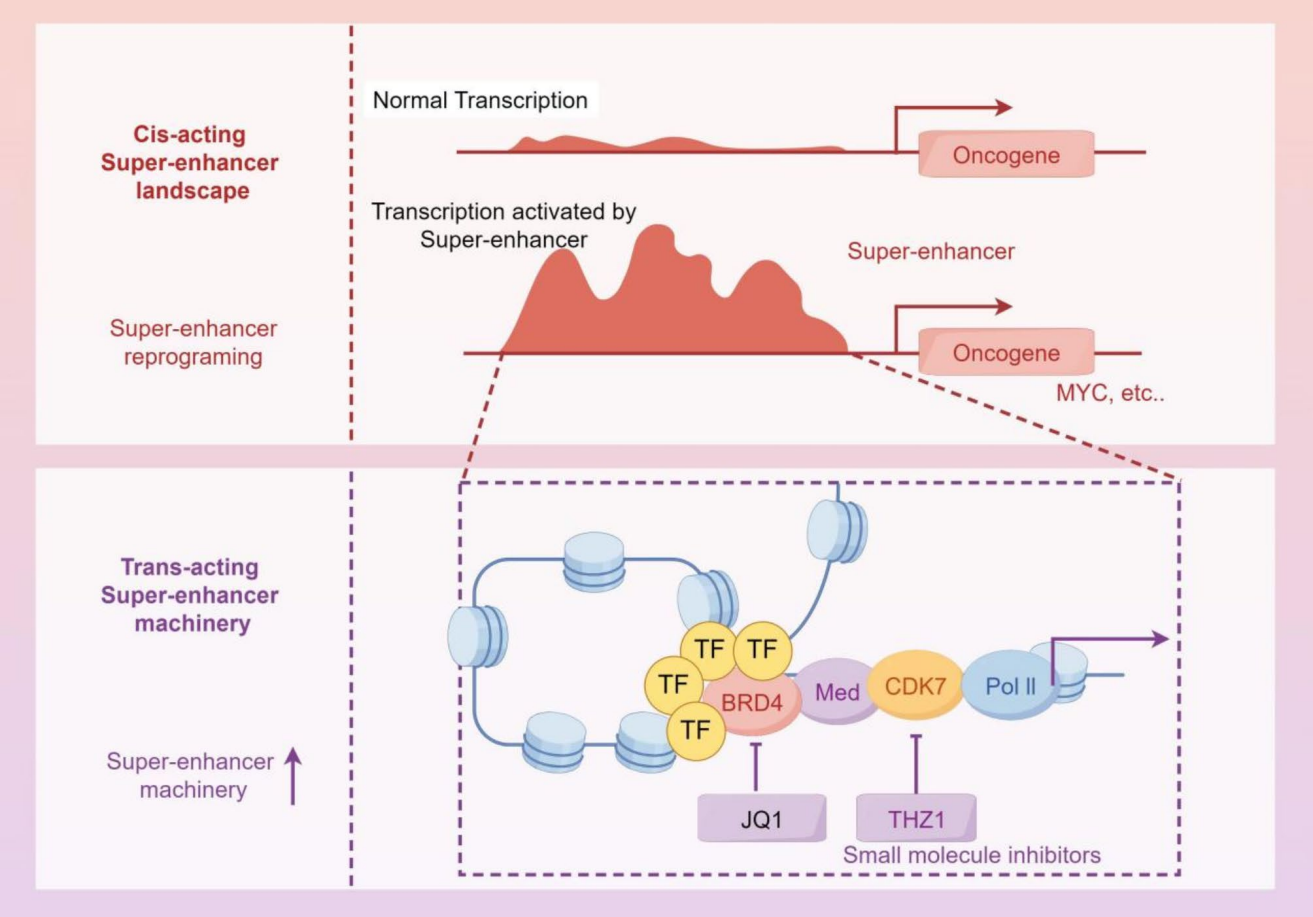
Schematic of super-enhancer structure in transcriptional activation Super enhancers drive transcription via promoter looping, recruiting factors to activate gene expression. JQ1 (a competitive inhibitor of BRD4) and THZ1 (a covalent inhibitor of CDK7 and CDK12) selectively kill cancer cells by inhibiting transcription of cancer-causing factors driven by super enhancers (By Figdraw)

The conventional pattern entails the attachment of a primary transcription factor (TF), which then enlists co-activating proteins including histone modifiers and ATP-dependent chromatin remodeling complexes [13]. The activation of the super enhancer facilitates a physical connection with the target promoter across extended distances, leading to the development of a looped DNA structure that brings the super enhancer and promoter into close spatial proximity. The chromatin loop facilitates contact between the RNA polymerase II transcription machinery and the target promoter, resulting in the activation of transcription [10, 46]. Fig. 2 presents the structural schematic of this conventional super - enhancer model, and some drugs can exert inhibitory effects on certain key proteins in this structure. The importance of this spatial interaction in promoting transcriptional activation is substantiated by evidence such as the β-globin promoter and enhancer. Despite being 40 kb apart, they are able to initiate transcription by forming a looping interaction. This establishes a direct connection between spatial interactions and transcriptional activation [47, 48].

Nevertheless, the specific methods and variables that facilitate the physical connection between enhancers and target promoters remain incompletely comprehended. Research has shown that architectural proteins, such CTCF, are more abundant at promoters and enhancers that are involved in chromatin looping [45]. The link between architectural proteins and mediator complexes has been established by immunoprecipitation tests. This suggests that these interactions can serve as a bridge between enhancers and promoters. Nevertheless, it is still uncertain whether there are any additional structural regulatory features that play a role in facilitating enhancer-promoter interactions. A study hypothesized that the transcription factor Yin Yang 1 (YY1) may facilitate physical contacts between enhancers and promoters, given that YY1 is a ubiquitously expressed protein capable of binding to enhancers and promoters in several cell types. Disruption of YY1 binding has been demonstrated to impede the connections between enhancers and promoters, consequently suppressing the production of the linked gene [49]. A burgeoning field of study is centered around enhancer RNA (eRNA), a recently identified element that has been linked to chromatin looping [50].Additional research is required to fully understand the mechanisms and variables that mediate enhancer-promoter interactions and the function of eRNA in chromatin looping.

### The phase-separation model

Furthermore, a novel phase separation model has been suggested for the assembly and activation of super enhancers, in addition to the conventional model of sequential recruitment of transcription factors and co-activators [51]. This paradigm is founded on the principle of phase separation, wherein dense clusters of proteins and nucleic acids, coupled with their mutually beneficial interactions, create organelles without membranes [52]. This process is widely recognized in the formation of ribonucleoprotein particles, such as processing bodies and stress granules [53, 54]. Super enhancers, which are regions of dense interactions between transcriptional regulators and nucleic acids, form quickly after a single initiation event [55] and disintegrate when the initiation site is removed [56, 57] or when high-density factors in the super enhancer region are depleted [16, 58–61]. This suggests that phase separation may play a role in the activation of super enhancers.

Providing evidence for this theory, a recent investigation revealed that transcription co-activators such BRD4 and MED1 attach to super enhancers in large quantities, creating clusters within the nucleus at the locations where super enhancer-mediated transcription occurs [62]. This phenomenon elucidates the heightened susceptibility of super enhancers to BET inhibitor medicines in contrast to traditional enhancers [51]. Additional investigation has revealed that the phase separation characteristics of BRD4 and MED1 are facilitated by their extensive disordered regions (IDRs). Some proteins that were previously found to have the ability to produce condensates share commonalities with these intrinsically disordered regions (IDRs) [63, 64]. Furthermore, it has been discovered that the activation domains of crucial transcription factors such as OCT4 and GCN4, along with the mediator complexes that are attached to super enhancers, are capable of initiating the creation of phase separation aggregates and performing a comparable function [65]. Some scholars have developed live-cell super-resolution and multi-color 3D-imaging approaches, and then proposed a three-way kissing model, which is also a supplement to the phase - separation model [66].These discoveries have presented a novel framework for regulating transcription through elements known as super enhancers, providing insights into the biological properties of super enhancers, including their formation, activation, and strong responsiveness to abundant co-activators [51].

However, recent reports also indicate that in the regulation of N - MYC expression, the phenomenon of phase separation affecting the expression level of N - MYC accounts for only a small proportion. Whether this phenomenon only exists in N - MYC expression or is more widespread requires more literature reports in the future [67].

## Super enhancers in different tumors

Super enhancers (SE) have been found in various tumor samples over the past decade after their discovery, and their downstream carcinogenic pathways and downstream factors are complex and diverse, which also expands our understanding of tumor pathogenesis and the search for more effective tumor treatment methods.

The FOSL1 protein in head and neck squamous cell carcinoma (HNSCC) interacts with specific mediator proteins, resulting in the formation of a functional complex known as a super enhancer. This intricate mechanism is involved in controlling the transcription of cancer-causing proteins, such as SNAI2 and FOSL1, which ultimately enhances the spread of HNSCC [68]. In addition, bromodomain protein 4 (BRD4) attracts mediators and NF-κB. Collectively, these genes combine to provide powerful enhancers on oncogenes including TP63, MET, and FOSL1, thereby exerting their functional impacts [69]. The long non-coding RNA TMEM44-AS1 directly interacts with SerpinB3 in glioma, a specific type of brain tumor. This interaction triggers the activation of the MYC and EGR1/IL-6 signaling pathways. MYC further interacts with MED1 and directly binds to the promoter and super enhancer regions of TMEM44-AS1, thereby promoting its transcription. This establishes a favorable cycle of interaction with TMEM44-AS1, which enhances the growth, infiltration, and movement of glioma cells [70]. Another study has similarly reported findings to those previously noted [17].

In 2021, Huang et al. provided evidence that the super enhancer is excessively active in triple-negative breast cancer (TNBC), facilitating the expression of FOXC1, MET, and ANLN genes. These genes play a role in facilitating tumor growth and are associated with a negative prognosis [71]. Similarly, Zheng et al. discovered that in breast cancer with estrogen receptor alpha-positive (ERα+) cells, super enhancers play a role in a beneficial feedback mechanism that controls the production of estrogen receptors. The activation of the RAS/RAF/MEK2/ERK/p90RSK/ERα phosphorylation cascade occurs through the involvement of BRD4 and RET, leading to tumor malignancy [72].Furthermore, there have been reports indicating that super enhancers play a role in promoting tumor growth in breast cancer through the regulation of long non-coding RNAs. The presence of these RNAs can be identified in both ER + and TNBC patients, serving as a diagnostic indicator for early diagnosis of breast cancer [73]. Furthermore, research on breast cancer has demonstrated that super enhancers stimulate the activation of PD-L1 and PD-L2 expression, consequently impacting the evasion of the immune system by tumor cells [74].

Bal et al. found that in diffuse large B-cell lymphoma (DLBCL), mutations in super enhancers associated with oncogenes BCL6, BCL2, and CXCR4 can hinder the interaction between inhibitory protein transcription (such as BLIMP1 for BCL6 and steroid receptors) and their respective target genes. Additionally, these mutations lead to a decrease in the transcription of NR3C1 for BCL2 and CXCR4 [75]. AML, a type of leukemia, has been found to be influenced by 200 oncogenes that are associated with super enhancers. These oncogenes play a crucial role in controlling the growth and survival of AML cells [76]. Studies have demonstrated that in multiple myeloma, super enhancers facilitate the excessive activation of the histone chaperone protein HJURP. Suppressing the expression of HJURP or reducing the activity of super enhancers can lead to a reduction in the survival of tumor cells and the initiation of programmed cell death, offering a promising new target for treating multiple myeloma [77]. RUNX3 interacts with upstream super enhancers in NKTL to control the transcription of TOX2. Increased expression of TOX2 can influence the growth, survival, ability to form colonies, and prognosis of tumor cells. Moreover, the phosphatase PRL-3, which is associated with the spread of cancer cells, has been recognized as a crucial protein controlled by TOX2 in the regulation of tumor growth [78].

Liang et al. reported in liver cancer that super enhancers affected the expression of non coding RNA lncRNA-DAW. Elevated lncRNA-DAW mediated the degradation of EZH2 (a negative regulatory factor of WNT2), and WNT2 activated Wnt/β-catenin pathway after it’s removed inhibition, which promotes the progression of liver cancer [79]. At the same time, activation of SIRT7 super enhancer was found in all samples in hepatocellular carcinoma, and it was found that SIRT7 and methyltransferase EZH2 were co expressed and bound in hepatocellular carcinoma. When SIRT7 super enhancer function was downregulated, it showed significant tumor suppressive activity [80]. In pancreatic ductal adenocarcinoma (PDAC), a super enhancer mediated RNA binding protein HNRNPF was reported to affect downstream PRMT1 and UBAP2L, promoting PDAC growth by enhancing mRNA translation [81].

In colorectal cancer, CDK12 regulates BCL2L1 and CCDC137 via super enhancers, increasing the risk of liver metastasis [82]. JQ1 and iBET-151 can inhibit colorectal cancer development, metastasis, and IL-20RA expression, demonstrating that IL-20RA, activated by super enhancers, affects colorectal cancer cell proliferation and immune escape [83].

Alam et al. reported that there was a widespread inactivation mutation of histone methyltransferase KMT2D in lung cancer, leading to impaired function of super enhancers and downregulation of PER2 (an inhibitor of glycolysis protein) expression, which promotes the glycolysis process in lung cancer cells and achieves the effect of promoting tumor growth [84]. In lung adenocarcinoma, M2 like tumor associated macrophages (TAM2) produced a TGF- β-rich microenvironment. The microenvironment activated SMAD3 to bind the promoter of long chain non coding RNA LINC01977 to the super enhancer, increasing the expression level of LINC01977. LINC01977 was also expressed through TGF- β/SMAD3 pathway to promote tumor development [85].

In ovarian cancer, two super enhancers were identified to have significant regulatory effects on cell proliferation, migration, and other functions [86]. Prostate cancer studies revealed that LSD1, along with BRD4 and FOXA1, formed a network enriched in the super enhancer region to regulate MYC expression and influence tumor development [87]. In bladder cancer, the super enhancer of SNHG15 was found to recruit FOSL1, thereby impacting tumor cell growth and metastasis through the WNT/CTNNB1 pathway [88].

Both in solid tumors and non - solid tumors, it has been found that super - enhancers play a role in promoting tumor development. However, there are differences in the regulatory factors that super - enhancers bind to and the genes whose expressions they regulate, which may affect multiple aspects of tumor functions including proliferation, migration, and invasion. Almost all super - enhancers discovered in tumors so far have promoted the malignant phenotypes of tumors. Therefore, based on the common features of super - enhancers functioning in different tumor cells, we can update our tumor diagnosis and treatment strategies. In particular, we can develop anti - cancer drugs applicable to multiple tumors by targeting specific targets. However, it has also been discovered that super - enhancers are capable of regulating certain tumor - suppressor genes within tumor cells. Nevertheless, these super - enhancers do not exert their functions in tumors [89–91]. Moreover, at present, evidence regarding the direct involvement of super - enhancers in tumor suppression is lacking, which demands further research on super - enhancers in tumors.

**Table 1:** Recent research highlights on the function of super-enhancers in tumorigenesis This form collates a subset of contemporary research findings on super enhancers in tumors, delineating the tumor types in which these enhancers operate, the interacting factors, the downstream signaling pathways, and their specific functional roles within the context of tumorigenesis

Tumor type	Binding factor	Downstream pathways/target genes	Function	Ref.
Squamous cell carcinoma of the head and neck (HNSCC)	FOSL1	SNAI2, FOSL1	Tumor metastasis	[68]
Squamous cell carcinoma of the head and neck (HNSCC)	BRD4, NF- κ B. P65	TP63, MET, FOSL1	Regulating tumor occurrence and growth	[69]
Glioma	Long chain non coding RNA TMEM44-AS1	Myc, EGR1/IL-6	Tumor cell growth, migration, and invasion	[70]
Triple negative breast cancer (TNBC)	FOXC1	MET, ANLN	Tumor growth and poor prognosis	[71]
ER α Positive breast cancer	BRD4, RET	RAS/RAF/MEK2/ERK/p90RSK	Malignant phenotype of tumors	[72]
Breast cancer	-	PD-L1、PD-L2	Immune escape of tumor cells	[74]
Diffuse large B-cell lymphoma (DLBCL)	-	BLIMP1, NR3C1	Tumor growth	[75]
Multiple myeloma	-	HJURP	Regulating tumor growth and survival	[77]
Natural Killer Cell/T-Cell Lymphoma (NKTL)	RUNX3	TOX2	Tumor cell proliferation, survival, colony forming ability, and prognosis of tumor patients	[78]
liver cancer	Non coding RNA lncRNA-DAW	Wnt/ β- Catenin	Tumor growth and progression	[79]
Hepatocellular carcinoma	-	SIRT7, EZH2	Growth and progression of tumors	[80]
Pancreatic Ductal Adenocarcinoma (PDAC)	-	HNRNP, FPRMT1,UBAP2L	Promoting tumor growth by enhancing mRNA translation	[81]
colorectal cancer	CDK12	BCL2L1, CCDC137	Liver metastasis of colorectal cancer	[82]
colorectal cancer	-	IL-20RA	Proliferation and immune escape of colorectal cancer cells	[83]
lung cancer	-	PER2	Promoting glycolysis and tumor cell growth	[84]
Lung adenocarcinoma	-	SMAD3, Long Chain Non coding RNA LINC01977	Tumor cell growth	[85]
prostatic cancer	LSD1, BRD4, FOXA1	MYC	Tumor growth	[87]
Bladder cancer	FOSL1	WNT/CTNNB1	Tumor growth and metastasis	[88]

## Research on the treatment of super enhancers in tumors

Today, we have developed various methods for the treatment of tumors, including surgical treatment, chemotherapy drug treatment, radiation therapy, immunotherapy, etc. However, each method has its own shortcomings and limitations. We need a broader mindset to strengthen our arsenal of weapons against tumors.

Following the establishment of the correlation between super enhancers and tumors, there has been a progressive development of tumor treatment strategies that specifically target super enhancers. Cancer cells generally demonstrate elevated levels of oncogenic gene transcription activity, which is not commonly observed in normal healthy cells [6, 92]. Hence, targeting the suppression of oncogenic factor transcription in tumor tissue is an optimal therapeutic approach, albeit a formidable hurdle in drug discovery. Targeted therapy aimed at modulating transcription, a fundamental biological process common to all living cells, has the potential to exert substantial influence on overall gene expression [6, 93]. Hence, it is imperative for any transcriptional inhibitor employed in clinical therapy to specifically target the suppression of oncogenic factor transcription while minimizing damage in normal cells. Recent research has shown that JQ1, a substance that competes with BRD4, and THZ1, a substance that forms a strong bond with CDK7 and CDK12, can effectively destroy cancer cells by blocking the process of transcribing oncogenic factors that are activated by super enhancers. Crucially, these medications do not have any harmful effects on the body as a whole, which makes them highly favorable options for treatment [58–60]. This text will present a comprehensive summary of the targeting principles and mechanisms of action of JQ1 and THZ1.

BRD4, a constituent of the bromodomain and extra terminal (BET) protein family, has been recognized as a pivotal factor in multiple human ailments, encompassing cancer, cardiovascular disease, inflammatory disease, and central nervous system (CNS) sickness [55, 69, 87, 94–98]. The process of transcription commencement, halt, and extension entails the synchronized substitution of regulatory factors and co-factors. Transcription facilitated by super enhancers (SE) depends on the cooperative interaction between BRD4 and mediator complexes, as well as the step-by-step recruitment of TFIIH initiation complexes containing CDK7 and p-TEFb extension complexes containing CDK9 [99]. RNA Pol II begins the process of transcription by phosphorylating CDK7 and promotes pausing of the proximal promoter by recruiting negative elongation factor (NELF) and DRB sensitivity-inducing factor (DSIF) to RNA Pol II [100]. The process of releasing transcriptional pauses and transitioning to productive elongation is facilitated by the phosphorylation of RNA Pol II and DSIF by the action of p-TEFb (CDK9) [99]. In addition, BRD4 plays a role in promoting the assembly of super-enhancers by beginning the recruitment of mediator complexes. It also actively facilitates the elongation of gene transcription mediated by super-enhancers by aiding in the release of pauses [101]. CDK12/13 are also engaged in elongation, whereas CDKs play a role in mRNA processing [102, 103]. Hence, the mediator complexes, key regulatory factors that exert influence on transcription, BRD4, and CDKs all serve as potential targets for cancer therapy by suppressing carcinogenic transcription.

In order to substantiate this viewpoint, a considerable number of tiny chemical compounds that block BRD4 have been created. Among these, JQ1 was the initial inhibitor of the BET family to be discovered and has been intensively researched. JQ1 specifically attaches to the acetyllysine recognition domain of BRD4, impeding the growth of tumors by restricting its chromatin-dependent role in regulating promoters and enhancers [103–105]. Aligned with the imbalanced distribution of BRD4 on super enhancers [16, 58] and its involvement in promoting super enhancer activity [101], treatment with JQ1 selectively reduces the levels of BRD4 and CDK9 at these locations, leading to the arrest of RNA Pol II and inhibition of elongation. The inhibitory effect is especially noticeable in preserving the cellular ability to differentiate into different cell types and the cancer-causing properties of the super enhancer-driven BRD4-dependent transcriptional programme [58, 101]. Simultaneously, research reports in diverse tumors have demonstrated that the application of BRD4 inhibitors can result in the reduction of H3K27ac and the increment of H3K27me3 at enhancer loci [106], alterations in advanced chromatin structure [107], suppression of SE - directed co - transcriptional pri - miRNA processing [108, 109], and among the genes, those regulated by super - enhancers are the most affected, for instance, MYC [16, 110]. This implies that the utilization of BRD4 inhibitors indeed exerts multi - aspect and far - reaching influences on the functions of super - enhancers within tumors. In diffuse large B-cell lymphoma, therapy with JQ1 results in the suppression of transcription of lineage-specific transcription factor genes and oncogenes mediated by super enhancers. This leads to a decrease in tumor growth and an improvement in patient survival [58]. The suppression of carcinogenic transcription mediated by super enhancers through BRD4 inhibition has been verified in other types of cancer as well [76, 111–113]. In the context of castration-resistant prostate cancer (CRPC), a study found that the suppression of tumor growth was notably increased when both lysine-specific demethylase (LSD1) and BET protein inhibitors were administered as part of the treatment [87].

The cooperative interaction between BRD4 and other chromatin regulatory proteins in facilitating SE-dependent transcription offers numerous possibilities for combination therapy. BRD4 inhibition in acute myeloid leukaemia (AML) results in the reduction of mediator complexes linked to super-enhancers (SEs) that control important leukaemia genes, hence inhibiting tumor growth [112]. The interaction between disruptor of telomere silencing 1-like (DOT1L)-mediated histone H3K79 methylation and histone H4 acetylation in MLL facilitates the binding of BRD4 to super-enhancers (SEs). The simultaneous blocking of DOT1L and BRD4 effectively hinders the growth and multiplication of leukaemia cells [114]. SE-mediated gene regulation also plays a role in the senescence-associated secretory phenotype (SASP), which is an immunological surveillance system that gets rid of pre-cancerous senescent cells [115]. The activation of the SASP gene expression programme entails the creation of novel BRD4-rich super-enhancers (SEs) in close proximity to SASP genes, while simultaneously repressing SEs linked to genes involved in proliferation [113]. When BRD4 is inhibited in living organisms, it leads to a decrease in the SASP transcriptional programme in cancer-causing N-RAS-expressing cells. This impairs the cells’ ability to be removed from the body [116].

The susceptibility of cancer cells to THZ1 further exemplifies the significant reliance of cancer cells on SE-driven transcription. THZ1 is a potent and selective covalent inhibitor of CDK7, with a limited inhibitory impact on CDK12 [58–61]. THZ1 suppresses the phosphorylation of the carboxyl terminal domain (CTD) of RNA Pol II, therefore impeding the immediate release of the promoter [100]. THZ1-induced pause deficits lead to a decrease in RNA Pol II occupancy on enhancers that require pause at SE. This ultimately results in transcriptional suppression [117, 118]. THZ1 therapy can cause a loss of SE function, resulting in a notable decrease in oncogenic transcription and suppression of tumor growth in different kinds of cancer [58–61].

THZ1 selectively targets the SE-dependent expression of particular subsets of cell type-specific key transcription factors (RUNX1, MYCN, and MYC) in all of these malignancies. These transcription factors play a significant role in regulating the core transcription programme that is important for sustaining the identity of tumor cells. The oncogene MYC, in particular, has been demonstrated to promote the growth and replication of cancer cells by amplifying and augmenting gene transcription. Hence, suppressing MYC activity could prove to be an exceedingly efficacious therapeutic approach. Neuroblastoma (NB) cells have been found to achieve genomic amplification of MYCN oncogenes by promoting super-enhancers (SE). This results in the upregulation of MYCN’s active transcription programme and increases the sensitivity of NB cells to CDK7 inhibition. THZ1, by selectively inhibiting CDK7, was utilized to treat MYCN amplified NB cells. This resulted in a specific decrease in SE associated genes and a notable suppression of MYCN SE dependent transcriptional amplification. Consequently, tumor development and proliferation were inhibited significantly [59].

Inhibiting CDK12 can effectively target SE-driven transcription, as CDK12 serves as a positive regulator of transcriptional elongation [82, 119]. Alternatively, SE function can be negatively regulated by inhibiting the mediator kinases CDK8/19 [120, 121]. Targeted inhibition of these mediator kinases in acute myeloid leukaemia (AML) results in the activation of tumor suppressor genes and lineage-specific transcription factors, ultimately leading to anti-leukemia actions [120]. In addition, Kennedy et al. discovered genes associated with super-enhancers (SEs) and provided insights on several underestimated carcinogenic genes. They specifically emphasised the significance of the SE-driven cyclin D1 site in Ewing’s sarcoma. Targeting this location may increase the likelihood of inhibiting cyclin D/CDK4 [122]. Furthermore, there has been a detection of excessive expression of ERG in both primary prostate cancer and castration-resistant prostate cancer (CRPC). NSC139021 has been found as a potent and specific small molecule inhibitor of ERG, significantly suppressing the proliferation of cancer cells that express ERG [123]. YK-4-279, a different type of substance that inhibits the activity of molecules, has been found to effectively decrease the growth of prostate cancer patient-derived xenografts that include the ERG protein [124].

**Table 2:** Partial compilation of therapeutic drugs targeting super-enhancers in tumors The tabular data provided here consolidates a selection of therapeutic agents and compounds directed against super enhancers in tumors, encompassing those at both preclinical and experimental stages. This compilation meticulously records the particular molecular targets engaged by these therapeutic strategies. A concise exposition is provided concerning the mechanisms by which these agents combat cancer, as well as specifying the types of cancer that are the primary focus of studies involving these drugs or compounds

SE inhibitor	Inhibition Target	Function	Tumor type	Ref.
JQ1	BRD4	By consuming BRD4 and CDK9, this results in the stalling and inhibition of Pol II elongation.	DLBCL, CRPC…	[58, 87]
THZ1	CDK7	Inhibiting the phosphorylation of the carboxyl-terminal domain (CTD) of RNA Polymerase II, thereby obstructing the proximal promoter pausing.	DLBCL, Neuroblastoma, T-ALL, Triple-negative breast cancer (TNBC)…	[58–61]
SR-4835, THZ531	CDK12	Suppression of CDK12-mediated phosphorylation of Serine 2 (S2) within the carboxyl-terminal domain (CTD) of RNA Polymerase II, thereby hindering transcriptional elongation.	Colorectal cancer (CRC)	[82, 119]
NSC139021,YK-4-279	ERG	Inhibition of ERG expression elicits suppression of tumor growth.	Primary prostate cancer and castration-resistant prostate cancer (CRPC)	[123, 124]

At now, it is unclear how SE-related genes are particularly sensitive to suppression of chromatin/transcriptional regulatory factors. One possible explanation is that cancer cells in multiple myeloma are more sensitive to JQ1 because it has a greater ability to bind to BRD4 and Mediator at specific places called SE sites. These SE sites are related with genes like MYC and other survival genes that are specific to the cancer type. JQ1 has a preference for occupying these sites [16]. The conclusion is further substantiated by the evidence of direct targeting of BET inhibitors on super-enhancers (SEs) [103]. However, a later investigation in AML cells shown that the removal of Mediator by JQ1 happened in less than 50% of all super-enhancers (SEs) linked to cancer-related genes. This expulsion was found to be independent of the initial levels of this transcription cofactor [112]. In contrast, the SE-related transcription programme that is most impacted by JQ1 demonstrates elevated levels of MYB binding capacity. Prior research has demonstrated that heightened susceptibility of SE-related genes to inhibitory influences can be ascribed to at least two complimentary mechanisms: the combined impact of component enhancers and the brief duration of carcinogenic transcription factors [58–61]. Notwithstanding these discoveries, there remains a dearth of definitive comprehension in this domain, necessitating additional research to properly grasp the function of super enhancers.

## The problems faced by super enhancer targeted therapy

While these inhibitors exhibit low systemic toxicity and high selectivity, they can nonetheless interfere with fundamental transcription mechanisms and ultimately impact overall gene expression. This matter demands attention, as nearly all human tissues possess SE that preserves tissue uniqueness for proper functioning. Simultaneously, several SE components may display varying susceptibilities to inhibition, contingent upon the binding of each component within the complex generated by SE during its operation. The degree of cellular reliance on SE through this binding also impacts the susceptibility to SE inhibition [20].

Regrettably, recent studies have discovered that the effectiveness of BRD4 targeted therapy is being hindered by the development of resistance. In breast cancer cells, researchers unexpectedly discovered that BRD4 interacts with the LSD1/NuRD complex to form an inhibitory complex, which co - localizes on super - enhancers. Simultaneously, the BRD4/LSD1/NuRD complex restricts the over - activation of a group of genes associated with drug - resistant functions. Long - term treatment with JQ1 or the destabilization of LSD1 induced by PELI1 will cause the BRD4/LSD1/NuRD complex to become ineffective, thereby leading to tumor resistance to JQ1 and broad - spectrum therapeutic compounds. It is also mentioned in the article that we may need to consider using the combined targeted chemotherapy of BRD4 and PELT1 to address this issue; however, certain clinical studies for verification are still lacking. The article also mentions that perhaps we need to consider adopting BRD4 and PELT1 - targeted chemotherapy in combination to deal with this problem, but it still lacks certain clinical studies for verification [90].

Moreover, the mechanisms through which tumors develop resistance to BRD4 are multifarious. It has been reported that cancer - associated fibroblasts (CAF) can induce the phosphorylation of BRD4 at tyrosine 97/98 in colorectal cancer via the interleukin − 6/8 - JAK2 transduction. Moreover, the interaction with the deubiquitinating enzyme UCHL3 results in the stabilization of BRD4 and a reduced binding capacity to BET inhibitors, thereby leading to tumor cell resistance. The inhibition of the IL6/IL8 - JAK2 signaling can eliminate BRD4 phosphorylation and restore the sensitivity of tumors to BET inhibitors [125].In KRAS - mutated tumors, including non - small - cell lung cancer (NSCLC), an up - regulation of BCL6 has been observed. BCL6 interacts with BRD3 and activates the mTOR signaling pathway, which promotes the resistance of tumors to BET inhibitors. The inhibition of BCL6 or mTOR can reverse the resistance of tumor cells [126].In addition, the activation of the MAPK signaling pathway [127], c – MYC [128], etc., also contributes to the induction of tumor resistance. Therefore, during the treatment, it is necessary to use the combination of BET inhibitors and inhibitors of other key targets to overcome the resistance problem.

Furthermore, studies have indicated that in the case of prostate cancer, JQ1 can enhance the invasion of tumors by suppressing FOXA1, a protein that inhibits invasion in prostate cancer. This is achieved by activating many pathways such as EMT, bone morphogenetic protein (BMP) signaling, chemokine signaling, and sticky plaques [129]. Similar findings have been reported in the treatment of B-cell lymphoma, where drugs specifically targeting SE (super-enhancer) effectively restrict the expression of oncogenes associated with SE, as well as the expression of tumor suppressor genes that depend on SE [31, 89]. Contrary to expectations, this anti-cancer medication actually exacerbates the negative consequences of tumor invasion, making it a topic of significant concern. Fortunately, the article has reported a BMP signaling inhibitor, LDN − 212,854. The application of this inhibitor can significantly suppress JQ1 - induced cell invasion and the expression of some genes in the EMT (epithelial - mesenchymal transition) pathway. In future cancer therapies, the combined treatment of JQ1 and BMP signaling inhibitors might be considered [129].

Scientists doing parallel research have found that inhibitors targeting SE-related processes may not exhibit therapeutic efficacy in certain subtypes of prostate cancer (PCa) [130, 131]. Cell lines and organoids derived from persons with SPOP mutations exhibited resistance to the inhibition of BRD4, resulting in the prevention of cell growth arrest and death [132, 133]. Nevertheless, it should be noted that BRD4 inhibitors are not entirely ineffective, and the resistance of SPOP mutant PCa to BRD4 inhibition can be surmounted by combining them with AKT inhibitors. Moreover, SPOP mutations can function as biomarkers for directing treatment strategies in prostate cancer patients, assessing the efficacy of this therapeutic approach, and facilitating the delivery of accurate medical care to patients [134].

While BET inhibitors, including JQ1, now show potential as cancer therapies, the emergence of side effects and drug resistance necessitates the development of effective solutions. Contemporary studies highlight the importance of integrating BET inhibitors with other cytotoxic medicines. For instance, the simultaneous application of BRD4 inhibitors and HDAC inhibitors has been shown to limit the growth of tumors and induce programmed cell death (apoptosis) in different tumor models, such as breast cancer, acute myeloid leukaemia, pancreatic ductal adenocarcinoma, neuroblastoma, and cutaneous T-cell lymphoma [135–140]. The BET inhibitor OTX015 and the proteasome inhibitor Caffezomycin synergistically induce apoptosis in TERT altered neuroblastoma cells [141]. An impactful synergistic effect was shown in the treatment of small cell lung cancer (SCLC) using the BET inhibitor ABBV-075 in combination with BCL2 inhibitors [142]. The concurrent administration of JQ1 and CDK7 inhibitor THZ1 hinders the growth and division of head and neck squamous cell carcinoma cells, leading to programmed cell death and cellular ageing [143]. The combination of BET inhibitors and LSD1 inhibitors substantially suppresses the proliferation of castration-resistant prostate cancer (CRPC), surpassing the efficacy of using a single inhibitor [87]. Our investigation demonstrates that JQ1 can effectively counteract the immunological resistance caused by CAR-T cell therapy in glioblastoma (GBM) [144]. In addition to the combination with BET inhibitors, the literature has documented the concurrent application of the CDK9 inhibitor AZD4573 in conjunction with PIM kinase or PI3K inhibitors in the context of diffuse large B-cell lymphoma (DLBCL) and primary lymphoma cells. Such combinatorial approaches have been demonstrated to effectively mitigate resistance to CDK9 inhibitors [18].

In a great number of literature reports, BRD4 inhibitors are capable of significantly enhancing the induction of homologous recombination (HR) deficiency by suppressing the expression of DNA topoisomerase II - binding protein 1 (TOPBP1), BRCA1, RAD51 and other genes involved in DNA replication and DNA - damage repair, thus augmenting the sensitivity of tumors to PARP1 inhibitors [145–147].As a result, the research regarding the combination of BRD4 inhibitors and PARP1 inhibitors is also a highly potential research direction in current drug development. Currently, numerous reports on relevant drug designs have been published [148–150]. Favorable treatment outcomes have been attained in pancreatic cancer and triple - negative breast cancer. These facts comprehensively demonstrate that while performing its own function of suppressing super - enhancers, BRD4 inhibitors can enhance the anti - tumor function of PARP1 inhibitors, compensating for the deficiency of single - target inhibitors in being prone to drug resistance, and thus possess broad development prospects.

As a strong contender to BET inhibitors in the future, BRD4 degraders have drawn increasing attention from researchers in recent years. With the advent of target protein degradation (TPD) technology, current drug research and development can target those that were traditionally regarded as “undruggable” targets and those inaccessible to small - molecule inhibitors [151, 152]. The drug development of BRD4 degraders is a popular research area in the clinical application of TPD technology. Related patents keep emerging, and compounds that have entered the clinical trial stage have been reported [153, 154].Some literature indicates that in acute myeloid leukemia (AML) and acute lymphoblastic leukemia (ALL), the BRD4 degrader dBET6 is a more effective anti - cancer drug than the traditional BRD4 inhibitor JQ1, and it can targetedly inhibit some signal pathways that induce tumor drug resistance [155]. Additionally, BRD4 degraders also influence the function of super – enhancers [156, 157]. This undoubtedly opens up a new area for finding substitutes for BRD4 inhibitors. However, most of the current research on BRD4 degraders has not yet entered the clinical trial stage, and numerous experiments are still required to verify its development into a mature anti - cancer drug and its market launch.

Furthermore, recent investigative findings indicate that indole-3-lactic acid (IPA), a compound produced by bacteria, can augment the efficacy of αPD-1 immunotherapy mediated by CD8 + T cells in the treatment of diverse neoplasms, including melanoma, breast cancer, and colorectal carcinoma. The underlying mechanism involves IPA elevating the levels of histone H3 lysine 27 acetylation at the enhancer site of the Tcf7 gene, thereby modulating the stemness program of CD8 + T cells and their differentiation into progenitor exhausted CD8 + T cells (T_pex_), which in turn limits tumor progression [158]. Furthermore, studies have indicated that the concurrent administration of CDK7 and CDK12 inhibitors can potently diminish resistance to hedgehog-targeted smoothened inhibitors (SMOi), thereby significantly augmenting the therapeutic efficacy of tumor-targeted therapies [159].These revelations suggest a novel hypothesis: whether super-enhancers could act as regulatory intermediaries in other therapeutic modalities, potentially being utilized as adjutant therapeutic agents within the clinical treatment paradigm.

## Conclusion

Over the past ten years, researchers have gained a fundamental understanding of super enhancers and gradually unraveled their involvement in tumorigenesis and progression. Currently, oncogenic super enhancers have been confirmed to exist in various cancer subtypes, playing a vital role in preserving the malignant characteristics of cells. By upregulating key oncogenes, super enhancers impart dependence on tumor-related genes, thereby providing a basis for identifying cancer targets with unclear driving genes. Research has shown that alterations in the structure and epigenetic characteristics of chromatin can result in changes to its three-dimensional arrangement. This can lead to the inappropriate activation of oncogenes through the hijacking of enhancers. Examining super enhancers within the framework of 3D chromatin tissue is crucial for a comprehensive understanding of their contribution to tumor formation.

From a therapeutic standpoint, the fundamental constituents of super enhancers are prevalent across several cancer subtypes. Individual constituents of super enhancers, such as BRD4, CDK7, or CDK9, have demonstrated significant potential in multiple preclinical tumor models. However, it cannot be ignored that the side effects of related drugs and their induced resistance remain a major challenge in our field of cancer treatment, as these drugs seem to be repeating the old path of traditional chemotherapy drugs. However, the combined application of these drugs and chemotherapy drugs targeting other targets, particularly in combination with PARP1 inhibitors, is a new hope for our future drug research and development. Therefore, more attention should be paid to finding more targeted treatment methods for specific tumors, and optimizing existing therapeutic methods.

Evidently, the research on super - enhancers in tumors is in its infancy. Future studies should center on further elucidating the mechanisms through which each component of super - enhancers modulates their functions. Additionally, a more in - depth understanding of the phase - separation patterns in the assembly and activation of super - enhancers may assist in uncovering the impacts of oncogenic signals on the assembly and functions of super - enhancers, thereby identifying novel potential drugs that are more specific to super - enhancers in cancer. This can minimize the emergence of side effects and tumor drug resistance as far as possible, making it a reliable and long - lasting treatment approach.

## Data Availability

All data generated or analyzed during this study are included in this published article.

## References

[1] Xia C, Dong X, Li H, Cao M, Sun D, He S, et al. Cancer statistics in China and United States, 2022: profiles, trends, and determinants. Chin Med J. 2022;135(5):584–90. 35143424 10.1097/CM9.0000000000002108 PMC8920425

[2] Bozic I, Allen B, Nowak MA. Dynamics of targeted cancer therapy. Trends Mol Med. 2012;18(6):311–6. 22595628 10.1016/j.molmed.2012.04.006 PMC3372676

[3] Nakhoda SK, Olszanski AJ. Addressing recent failures in Immuno-Oncology trials to Guide Novel Immunotherapeutic Treatment Strategies. Pharm Med. 2020;34(2):83–91. 10.1007/s40290-020-00326-z PMC8864519 32157638

[4] Bai R, Chen N, Li L, Du N, Bai L, Lv Z, et al. Mechanisms of Cancer Resistance to Immunotherapy. Front Oncol. 2020;10:1290. 32850400 10.3389/fonc.2020.01290 PMC7425302

[5] Hanahan D, Weinberg RA. Hallmarks of cancer: the next generation. Cell. 2011;144(5):646–74. 21376230 10.1016/j.cell.2011.02.013

[6] Bradner JE, Hnisz D, Young RA. Transcriptional addiction in Cancer. Cell. 2017;168(4):629–43. 28187285 10.1016/j.cell.2016.12.013 PMC5308559

[7] Lee TI, Young RA. Transcriptional regulation and its misregulation in disease. Cell. 2013;152(6):1237–51. 23498934 10.1016/j.cell.2013.02.014 PMC3640494

[8] Morgan MA, Shilatifard A. Chromatin signatures of cancer. Genes Dev. 2015;29(3):238–49. 25644600 10.1101/gad.255182.114 PMC4318141

[9] Buecker C, Wysocka J. Enhancers as information integration hubs in development: lessons from genomics. Trends Genet. 2012;28(6):276–84. 22487374 10.1016/j.tig.2012.02.008 PMC5064438

[10] Spitz F, Furlong EE. Transcription factors: from enhancer binding to developmental control. Nat Rev Genet. 2012;13(9):613–26. 22868264 10.1038/nrg3207

[11] Sur I, Taipale J. The role of enhancers in cancer. Nat Rev Cancer. 2016;16(8):483–93. 27364481 10.1038/nrc.2016.62

[12] Banerji J, Rusconi S, Schaffner W. Expression of a beta-globin gene is enhanced by remote SV40 DNA sequences. Cell. 1981;27(2 Pt 1):299–308. 6277502 10.1016/0092-8674(81)90413-x

[13] Calo E, Wysocka J. Modification of enhancer chromatin: what, how, and why? Mol Cell. 2013;49(5):825–37. 23473601 10.1016/j.molcel.2013.01.038 PMC3857148

[14] Shen H, Xu W, Guo R, Rong B, Gu L, Wang Z, et al. Suppression of enhancer overactivation by a RACK7-Histone demethylase complex. Cell. 2016;165(2):331–42. 27058665 10.1016/j.cell.2016.02.064 PMC4826479

[15] Hnisz D, Abraham BJ, Lee TI, Lau A, Saint-André V, Sigova AA, et al. Super-enhancers in the control of cell identity and disease. Cell. 2013;155(4):934–47. 24119843 10.1016/j.cell.2013.09.053 PMC3841062

[16] Lovén J, Hoke HA, Lin CY, Lau A, Orlando DA, Vakoc CR, et al. Selective inhibition of tumor oncogenes by disruption of super-enhancers. Cell. 2013;153(2):320–34. 23582323 10.1016/j.cell.2013.03.036 PMC3760967

[17] Chen Z, Tian D, Chen X, Cheng M, Xie H, Zhao J, et al. Super-enhancer-driven lncRNA LIMD1-AS1 activated by CDK7 promotes glioma progression. Cell Death Dis. 2023;14(6):383. 37385987 10.1038/s41419-023-05892-z PMC10310775

[18] Thieme E, Bruss N, Sun D, Dominguez EC, Coleman D, Liu T, et al. CDK9 inhibition induces epigenetic reprogramming revealing strategies to circumvent resistance in lymphoma. Mol Cancer. 2023;22(1):64. 36998071 10.1186/s12943-023-01762-6 PMC10061728

[19] Whyte WA, Orlando DA, Hnisz D, Abraham BJ, Lin CY, Kagey MH, et al. Master transcription factors and mediator establish super-enhancers at key cell identity genes. Cell. 2013;153(2):307–19. 23582322 10.1016/j.cell.2013.03.035 PMC3653129

[20] Sengupta S, George RE. Super-enhancer-driven Transcriptional dependencies in Cancer. Trends cancer. 2017;3(4):269–81. 28718439 10.1016/j.trecan.2017.03.006 PMC5546010

[21] Yoshino S, Suzuki HI. The molecular understanding of super-enhancer dysregulation in cancer. Nagoya J Med Sci. 2022;84(2):216–29. 35967935 10.18999/nagjms.84.2.216 PMC9350580

[22] Huang Y, Mouttet B, Warnatz HJ, Risch T, Rietmann F, Frommelt F, et al. The leukemogenic TCF3-HLF complex rewires Enhancers Driving Cellular Identity and Self-Renewal conferring EP300 vulnerability. Cancer Cell. 2019;36(6):630–e449. 31735627 10.1016/j.ccell.2019.10.004

[23] Thirant C, Ignacimouttou C, Lopez CK, Diop M, Le Mouël L, Thiollier C, et al. ETO2-GLIS2 hijacks transcriptional complexes to Drive Cellular Identity and Self-Renewal in Pediatric Acute Megakaryoblastic Leukemia. Cancer Cell. 2017;31(3):452–65. 28292442 10.1016/j.ccell.2017.02.006

[24] Terlecki-Zaniewicz S, Humer T, Eder T, Schmoellerl J, Heyes E, Manhart G, et al. Biomolecular condensation of NUP98 fusion proteins drives leukemogenic gene expression. Nat Struct Mol Biol. 2021;28(2):190–201. 33479542 10.1038/s41594-020-00550-w PMC7116736

[25] Ahn JH, Davis ES, Daugird TA, Zhao S, Quiroga IY, Uryu H, et al. Phase separation drives aberrant chromatin looping and cancer development. Nature. 2021;595(7868):591–5. 34163069 10.1038/s41586-021-03662-5 PMC8647409

[26] Pulikkan JA, Hegde M, Ahmad HM, Belaghzal H, Illendula A, Yu J, et al. CBFβ-SMMHC inhibition triggers apoptosis by disrupting MYC Chromatin Dynamics in Acute myeloid leukemia. Cell. 2018;174(1):172–e8621. 29958106 10.1016/j.cell.2018.05.048 PMC6211564

[27] Zhen T, Cao Y, Ren G, Zhao L, Hyde RK, Lopez G, et al. RUNX1 and CBFβ-SMMHC transactivate target genes together in abnormal myeloid progenitors for leukemia development. Blood. 2020;136(21):2373–85. 32929473 10.1182/blood.2020007747 PMC7685208

[28] Sun Y, Zhou B, Mao F, Xu J, Miao H, Zou Z, et al. HOXA9 reprograms the enhancer Landscape to Promote Leukemogenesis. Cancer Cell. 2018;34(4):643–e585. 30270123 10.1016/j.ccell.2018.08.018 PMC6179449

[29] Yoshino S, Yokoyama T, Sunami Y, Takahara T, Nakamura A, Yamazaki Y, et al. Trib1 promotes acute myeloid leukemia progression by modulating the transcriptional programs of Hoxa9. Blood. 2021;137(1):75–88. 32730594 10.1182/blood.2019004586 PMC7976434

[30] Vanden Bempt M, Demeyer S, Broux M, De Bie J, Bornschein S, Mentens N, et al. Cooperative enhancer activation by TLX1 and STAT5 drives development of NUP214-ABL1/TLX1-Positive T cell Acute Lymphoblastic Leukemia. Cancer Cell. 2018;34(2):271–. – 85.e7. 30107177 10.1016/j.ccell.2018.07.007 PMC6097876

[31] Zhang J, Vlasevska S, Wells VA, Nataraj S, Holmes AB, Duval R, et al. The CREBBP acetyltransferase is a haploinsufficient tumor suppressor in B-cell lymphoma. Cancer Discov. 2017;7(3):322–37. 28069569 10.1158/2159-8290.CD-16-1417 PMC5386396

[32] Brescia P, Schneider C, Holmes AB, Shen Q, Hussein S, Pasqualucci L, et al. MEF2B instructs Germinal Center Development and acts as an Oncogene in B Cell Lymphomagenesis. Cancer Cell. 2018;34(3):453–e659. 30205047 10.1016/j.ccell.2018.08.006 PMC6223119

[33] Wang C, Jiang S, Zhang L, Li D, Liang J, Narita Y et al. TAF Family proteins and MEF2C are essential for Epstein-Barr Virus Super-enhancer Activity. J Virol. 2019;93(16). 10.1128/JVI.00513-19 PMC6675876 31167905

[34] Manzano M, Günther T, Ju H, Nicholas J, Bartom ET, Grundhoff A et al. Kaposi’s Sarcoma-Associated Herpesvirus Drives a Super-Enhancer-Mediated Survival Gene Expression Program in Primary Effusion Lymphoma. mBio. 2020;11(4). 10.1128/mBio.01457-20 PMC7448273 32843547

[35] Gröschel S, Sanders MA, Hoogenboezem R, de Wit E, Bouwman BAM, Erpelinck C, et al. A single oncogenic enhancer rearrangement causes concomitant EVI1 and GATA2 deregulation in leukemia. Cell. 2014;157(2):369–81. 24703711 10.1016/j.cell.2014.02.019

[36] Yamazaki H, Suzuki M, Otsuki A, Shimizu R, Bresnick EH, Engel JD, et al. A remote GATA2 hematopoietic enhancer drives leukemogenesis in inv(3)(q21;q26) by activating EVI1 expression. Cancer Cell. 2014;25(4):415–27. 24703906 10.1016/j.ccr.2014.02.008 PMC4012341

[37] Northcott PA, Lee C, Zichner T, Stütz AM, Erkek S, Kawauchi D, et al. Enhancer hijacking activates GFI1 family oncogenes in medulloblastoma. Nature. 2014;511(7510):428–34. 25043047 10.1038/nature13379 PMC4201514

[38] Drier Y, Cotton MJ, Williamson KE, Gillespie SM, Ryan RJ, Kluk MJ, et al. An oncogenic MYB feedback loop drives alternate cell fates in adenoid cystic carcinoma. Nat Genet. 2016;48(3):265–72. 26829750 10.1038/ng.3502 PMC4767593

[39] Yun JW, Yang L, Park HY, Lee CW, Cha H, Shin HT, et al. Dysregulation of cancer genes by recurrent intergenic fusions. Genome Biol. 2020;21(1):166. 32631391 10.1186/s13059-020-02076-2 PMC7339451

[40] Ryan RJ, Drier Y, Whitton H, Cotton MJ, Kaur J, Issner R, et al. Detection of enhancer-Associated Rearrangements reveals mechanisms of Oncogene Dysregulation in B-cell lymphoma. Cancer Discov. 2015;5(10):1058–71. 26229090 10.1158/2159-8290.CD-15-0370 PMC4592453

[41] Mansour MR, Abraham BJ, Anders L, Berezovskaya A, Gutierrez A, Durbin AD, et al. Oncogene regulation. An oncogenic super-enhancer formed through somatic mutation of a noncoding intergenic element. Sci (New York NY). 2014;346(6215):1373–7. 10.1126/science.1259037 PMC4720521 25394790

[42] Elliott K, Larsson E. Non-coding driver mutations in human cancer. Nat Rev Cancer. 2021;21(8):500–9. 34230647 10.1038/s41568-021-00371-z

[43] Hnisz D, Weintraub AS, Day DS, Valton AL, Bak RO, Li CH, et al. Activation of proto-oncogenes by disruption of chromosome neighborhoods. Sci (New York NY). 2016;351(6280):1454–8. 10.1126/science.aad9024 PMC4884612 26940867

[44] Li K, Zhang Y, Liu X, Liu Y, Gu Z, Cao H, et al. Noncoding variants connect enhancer dysregulation with nuclear receptor signaling in hematopoietic malignancies. Cancer Discov. 2020;10(5):724–45. 32188707 10.1158/2159-8290.CD-19-1128 PMC7196497

[45] Kagey MH, Newman JJ, Bilodeau S, Zhan Y, Orlando DA, van Berkum NL, et al. Mediator and cohesin connect gene expression and chromatin architecture. Nature. 2010;467(7314):430–5. 20720539 10.1038/nature09380 PMC2953795

[46] Levine M, Cattoglio C, Tjian R. Looping back to Leap forward: transcription enters a new era. Cell. 2014;157(1):13–25. 24679523 10.1016/j.cell.2014.02.009 PMC4059561

[47] Deng W, Lee J, Wang H, Miller J, Reik A, Gregory PD, et al. Controlling long-range genomic interactions at a native locus by targeted tethering of a looping factor. Cell. 2012;149(6):1233–44. 22682246 10.1016/j.cell.2012.03.051 PMC3372860

[48] Chen H, Levo M, Barinov L, Fujioka M, Jaynes JB, Gregor T. Dynamic interplay between enhancer-promoter topology and gene activity. Nat Genet. 2018;50(9):1296–303. 30038397 10.1038/s41588-018-0175-z PMC6119122

[49] Weintraub AS, Li CH, Zamudio AV, Sigova AA, Hannett NM, Day DS, et al. YY1 is a Structural Regulator of enhancer-promoter loops. Cell. 2017;171(7):1573–e8828. 29224777 10.1016/j.cell.2017.11.008 PMC5785279

[50] Li W, Notani D, Ma Q, Tanasa B, Nunez E, Chen AY, et al. Functional roles of enhancer RNAs for oestrogen-dependent transcriptional activation. Nature. 2013;498(7455):516–20. 23728302 10.1038/nature12210 PMC3718886

[51] Hnisz D, Shrinivas K, Young RA, Chakraborty AK, Sharp PA. A phase separation model for Transcriptional Control. Cell. 2017;169(1):13–23. 28340338 10.1016/j.cell.2017.02.007 PMC5432200

[52] Bergeron-Sandoval LP, Safaee N, Michnick SW. Mechanisms and consequences of Macromolecular phase separation. Cell. 2016;165(5):1067–79. 27203111 10.1016/j.cell.2016.05.026

[53] Brangwynne CP, Eckmann CR, Courson DS, Rybarska A, Hoege C, Gharakhani J, et al. Germline P granules are liquid droplets that localize by controlled dissolution/condensation. Sci (New York NY). 2009;324(5935):1729–32. 10.1126/science.1172046 19460965

[54] Molliex A, Temirov J, Lee J, Coughlin M, Kanagaraj AP, Kim HJ, et al. Phase separation by low complexity domains promotes stress granule assembly and drives pathological fibrillization. Cell. 2015;163(1):123–33. 26406374 10.1016/j.cell.2015.09.015 PMC5149108

[55] Brown JD, Lin CY, Duan Q, Griffin G, Federation A, Paranal RM, et al. NF-κB directs dynamic super enhancer formation in inflammation and atherogenesis. Mol Cell. 2014;56(2):219–31. 25263595 10.1016/j.molcel.2014.08.024 PMC4224636

[56] Hnisz D, Schuijers J, Lin CY, Weintraub AS, Abraham BJ, Lee TI, et al. Convergence of developmental and oncogenic signaling pathways at transcriptional super-enhancers. Mol Cell. 2015;58(2):362–70. 25801169 10.1016/j.molcel.2015.02.014 PMC4402134

[57] Shin HY, Willi M, HyunYoo K, Zeng X, Wang C, Metser G, et al. Hierarchy within the mammary STAT5-driven Wap super-enhancer. Nat Genet. 2016;48(8):904–11. 27376239 10.1038/ng.3606 PMC4963296

[58] Chapuy B, McKeown MR, Lin CY, Monti S, Roemer MG, Qi J, et al. Discovery and characterization of super-enhancer-associated dependencies in diffuse large B cell lymphoma. Cancer Cell. 2013;24(6):777–90. 24332044 10.1016/j.ccr.2013.11.003 PMC4018722

[59] Chipumuro E, Marco E, Christensen CL, Kwiatkowski N, Zhang T, Hatheway CM, et al. CDK7 inhibition suppresses super-enhancer-linked oncogenic transcription in MYCN-driven cancer. Cell. 2014;159(5):1126–39. 25416950 10.1016/j.cell.2014.10.024 PMC4243043

[60] Kwiatkowski N, Zhang T, Rahl PB, Abraham BJ, Reddy J, Ficarro SB, et al. Targeting transcription regulation in cancer with a covalent CDK7 inhibitor. Nature. 2014;511(7511):616–20. 25043025 10.1038/nature13393 PMC4244910

[61] Wang Y, Zhang T, Kwiatkowski N, Abraham BJ, Lee TI, Xie S, et al. CDK7-dependent transcriptional addiction in triple-negative breast cancer. Cell. 2015;163(1):174–86. 26406377 10.1016/j.cell.2015.08.063 PMC4583659

[62] Sabari BR, Dall’Agnese A, Boija A, Klein IA, Coffey EL, Shrinivas K, et al. Coactivator condensation at super-enhancers links phase separation and gene control. Volume 361. New York, NY: Science; 2018. 6400. 10.1126/science.aar3958 PMC6092193 29930091

[63] Banani SF, Lee HO, Hyman AA, Rosen MK. Biomolecular condensates: organizers of cellular biochemistry. Nat Rev Mol Cell Biol. 2017;18(5):285–98. 28225081 10.1038/nrm.2017.7 PMC7434221

[64] Shin Y, Brangwynne CP. Liquid phase condensation in cell physiology and disease. Volume 357. New York, NY: Science; 2017. 6357. 10.1126/science.aaf4382 28935776

[65] Boija A, Klein IA, Sabari BR, Dall’Agnese A, Coffey EL, Zamudio AV, et al. Transcription factors activate genes through the phase-separation capacity of their activation domains. Cell. 2018;175(7):1842–e5516. 30449618 10.1016/j.cell.2018.10.042 PMC6295254

[66] Du M, Stitzinger SH, Spille JH, Cho WK, Lee C, Hijaz M, et al. Direct observation of a condensate effect on super-enhancer controlled gene bursting. Cell. 2024;187(2):331–. – 44.e17. 38194964 10.1016/j.cell.2023.12.005

[67] Yang J, Chung CI, Koach J, Liu H, Navalkar A, He H, et al. MYC phase separation selectively modulates the transcriptome. Nat Struct Mol Biol. 2024;31(10):1567–79. 38811792 10.1038/s41594-024-01322-6 PMC11479839

[68] Zhang M, Hoyle RG, Ma Z, Sun B, Cai W, Cai H, et al. FOSL1 promotes metastasis of head and neck squamous cell carcinoma through super-enhancer-driven transcription program. Mol Therapy: J Am Soc Gene Therapy. 2021;29(8):2583–600. 10.1016/j.ymthe.2021.03.024 PMC8353207 33794365

[69] Dong J, Li J, Li Y, Ma Z, Yu Y, Wang CY. Transcriptional super-enhancers control cancer stemness and metastasis genes in squamous cell carcinoma. Nat Commun. 2021;12(1):3974. 34172737 10.1038/s41467-021-24137-1 PMC8233332

[70] Bian E, Chen X, Cheng L, Cheng M, Chen Z, Yue X, et al. Super-enhancer-associated TMEM44-AS1 aggravated glioma progression by forming a positive feedback loop with Myc. J Experimental Clin cancer Research: CR. 2021;40(1):337. 10.1186/s13046-021-02129-9 PMC8543865 34696771

[71] Huang H, Hu J, Maryam A, Huang Q, Zhang Y, Ramakrishnan S, et al. Defining super-enhancer landscape in triple-negative breast cancer by multiomic profiling. Nat Commun. 2021;12(1):2242. 33854062 10.1038/s41467-021-22445-0 PMC8046763

[72] Zheng ZZ, Xia L, Hu GS, Liu JY, Hu YH, Chen YJ, et al. Super-enhancer-controlled positive feedback loop BRD4/ERα-RET-ERα promotes ERα-positive breast cancer. Nucleic Acids Res. 2022;50(18):10230–48. 36124682 10.1093/nar/gkac778 PMC9561272

[73] Ropri AS, DeVaux RS, Eng J, Chittur SV, Herschkowitz JI. Cis-acting super-enhancer lncRNAs as biomarkers to early-stage breast cancer. Breast cancer Research: BCR. 2021;23(1):101. 34717732 10.1186/s13058-021-01479-8 PMC8557595

[74] Xu Y, Wu Y, Zhang S, Ma P, Jin X, Wang Z, et al. A tumor-specific Super-enhancer drives Immune Evasion by Guiding Synchronous expression of PD-L1 and PD-L2. Cell Rep. 2019;29(11):3435–e474. 31825827 10.1016/j.celrep.2019.10.093

[75] Bal E, Kumar R, Hadigol M, Holmes AB, Hilton LK, Loh JW, et al. Super-enhancer hypermutation alters oncogene expression in B cell lymphoma. Nature. 2022;607(7920):808–15. 35794478 10.1038/s41586-022-04906-8 PMC9583699

[76] Fang F, Lu J, Sang X, Tao YF, Wang JW, Zhang ZM, et al. Super-enhancer profiling identifies novel critical and targetable cancer survival gene LYL1 in pediatric acute myeloid leukemia. J Experimental Clin cancer Research: CR. 2022;41(1):225. 10.1186/s13046-022-02428-9 PMC9288051 35842703

[77] Jia Y, Zhou J, Tan TK, Chung TH, Chen Y, Chooi JY, et al. Super enhancer-mediated Upregulation of HJURP promotes growth and survival of t(4;14)-Positive multiple myeloma. Cancer Res. 2022;82(3):406–18. 34893510 10.1158/0008-5472.CAN-21-0921 PMC9397631

[78] Zhou J, Toh SH, Tan TK, Balan K, Lim JQ, Tan TZ, et al. Super-enhancer-driven TOX2 mediates oncogenesis in natural Killer/T cell lymphoma. Mol Cancer. 2023;22(1):69. 37032358 10.1186/s12943-023-01767-1 PMC10084643

[79] Liang W, Shi C, Hong W, Li P, Zhou X, Fu W, et al. Super-enhancer-driven lncRNA-DAW promotes liver cancer cell proliferation through activation of Wnt/β-catenin pathway. Mol Therapy Nucleic Acids. 2021;26:1351–63. 10.1016/j.omtn.2021.10.028 PMC8608597 34853732

[80] Wu F, Xu L, Tu Y, Cheung OK, Szeto LL, Mok MT, et al. Sirtuin 7 super-enhancer drives epigenomic reprogramming in hepatocarcinogenesis. Cancer Lett. 2022;525:115–30. 34736960 10.1016/j.canlet.2021.10.039

[81] Antal CE, Oh TG, Aigner S, Luo EC, Yee BA, Campos T, et al. A super-enhancer-regulated RNA-binding protein cascade drives pancreatic cancer. Nat Commun. 2023;14(1):5195. 37673892 10.1038/s41467-023-40798-6 PMC10482938

[82] Dai W, Wu J, Peng X, Hou W, Huang H, Cheng Q, et al. CDK12 orchestrates super-enhancer-associated CCDC137 transcription to direct hepatic metastasis in colorectal cancer. Clin Translational Med. 2022;12(10):e1087. 10.1002/ctm2.1087 PMC9577262 36254394

[83] Yu D, Yang X, Lin J, Cao Z, Lu C, Yang Z, et al. Super-enhancer Induced IL-20RA promotes Proliferation/Metastasis and Immune Evasion in Colorectal Cancer. Front Oncol. 2021;11:724655. 34336707 10.3389/fonc.2021.724655 PMC8319729

[84] Alam H, Tang M, Maitituoheti M, Dhar SS, Kumar M, Han CY, et al. KMT2D Deficiency impairs super-enhancers to Confer a glycolytic vulnerability in Lung Cancer. Cancer Cell. 2020;37(4):599–e6177. 32243837 10.1016/j.ccell.2020.03.005 PMC7178078

[85] Zhang T, Xia W, Song X, Mao Q, Huang X, Chen B, et al. Super-enhancer hijacking LINC01977 promotes malignancy of early-stage lung adenocarcinoma addicted to the canonical TGF-β/SMAD3 pathway. J Hematol Oncol. 2022;15(1):114. 35982471 10.1186/s13045-022-01331-2 PMC9389757

[86] Kelly MR, Wisniewska K, Regner MJ, Lewis MW, Perreault AA, Davis ES, et al. A multi-omic dissection of super-enhancer driven oncogenic gene expression programs in ovarian cancer. Nat Commun. 2022;13(1):4247. 35869079 10.1038/s41467-022-31919-8 PMC9307778

[87] Li M, Liu M, Han W, Wang Z, Han D, Patalano S, et al. LSD1 inhibition disrupts Super-enhancer-driven Oncogenic Transcriptional Programs in Castration-resistant prostate Cancer. Cancer Res. 2023;83(10):1684–98. 36877164 10.1158/0008-5472.CAN-22-2433 PMC10192194

[88] Tan M, Pan Q, Gong H, Zhai X, Wan Z, Ge M, et al. Super-enhancer-associated SNHG15 cooperating with FOSL1 contributes to bladder cancer progression through the WNT pathway. Pharmacol Res. 2023;197:106940. 37758102 10.1016/j.phrs.2023.106940

[89] Ding Y, Zhang B, Payne JL, Song C, Ge Z, Gowda C, et al. Ikaros tumor suppressor function includes induction of active enhancers and super-enhancers along with pioneering activity. Leukemia. 2019;33(11):2720–31. 31073152 10.1038/s41375-019-0474-0 PMC6842075

[90] Liu B, Liu X, Han L, Chen X, Wu X, Wu J et al. BRD4-directed super-enhancer organization of transcription repression programs links to chemotherapeutic efficacy in breast cancer. Proc Natl Acad Sci USA. 2022;119(6). 10.1073/pnas.2109133119 PMC8832982 35105803

[91] Deng R, Huang JH, Wang Y, Zhou LH, Wang ZF, Hu BX, et al. Disruption of super-enhancer-driven tumor suppressor gene RCAN1.4 expression promotes the malignancy of breast carcinoma. Mol Cancer. 2020;19(1):122. 32771023 10.1186/s12943-020-01236-z PMC7414732

[92] Lin CY, Lovén J, Rahl PB, Paranal RM, Burge CB, Bradner JE, et al. Transcriptional amplification in tumor cells with elevated c-Myc. Cell. 2012;151(1):56–67. 23021215 10.1016/j.cell.2012.08.026 PMC3462372

[93] Bhagwat AS, Vakoc CR. Targeting transcription factors in Cancer. Trends cancer. 2015;1(1):53–65. 26645049 10.1016/j.trecan.2015.07.001 PMC4669894

[94] Qin ZY, Wang T, Su S, Shen LT, Zhu GX, Liu Q, et al. BRD4 promotes gastric Cancer Progression and Metastasis through Acetylation-Dependent stabilization of snail. Cancer Res. 2019;79(19):4869–81. 31311807 10.1158/0008-5472.CAN-19-0442

[95] Shi C, Ye Z, Han J, Ye X, Lu W, Ji C, et al. BRD4 as a therapeutic target for nonfunctioning and growth hormone pituitary adenoma. Neurooncology. 2020;22(8):1114–25. 10.1093/neuonc/noaa084 PMC7594556 32246150

[96] Anand P, Brown JD, Lin CY, Qi J, Zhang R, Artero PC, et al. BET bromodomains mediate transcriptional pause release in heart failure. Cell. 2013;154(3):569–82. 23911322 10.1016/j.cell.2013.07.013 PMC4090947

[97] Kim SY, Zhang X, Schiattarella GG, Altamirano F, Ramos TAR, French KM, et al. Epigenetic reader BRD4 (bromodomain-Containing protein 4) governs nucleus-encoded mitochondrial transcriptome to regulate cardiac function. Circulation. 2020;142(24):2356–70. 33113340 10.1161/CIRCULATIONAHA.120.047239 PMC7736324

[98] Liang D, Yu Y, Ma Z. Novel strategies targeting bromodomain-containing protein 4 (BRD4) for cancer drug discovery. Eur J Med Chem. 2020;200:112426. 32502863 10.1016/j.ejmech.2020.112426

[99] Adelman K, Lis JT. Promoter-proximal pausing of RNA polymerase II: emerging roles in metazoans. Nat Rev Genet. 2012;13(10):720–31. 22986266 10.1038/nrg3293 PMC3552498

[100] Nilson KA, Guo J, Turek ME, Brogie JE, Delaney E, Luse DS, et al. THZ1 reveals roles for Cdk7 in co-transcriptional capping and pausing. Mol Cell. 2015;59(4):576–87. 26257281 10.1016/j.molcel.2015.06.032 PMC4546572

[101] Di Micco R, Fontanals-Cirera B, Low V, Ntziachristos P, Yuen SK, Lovell CD, et al. Control of embryonic stem cell identity by BRD4-dependent transcriptional elongation of super-enhancer-associated pluripotency genes. Cell Rep. 2014;9(1):234–47. 25263550 10.1016/j.celrep.2014.08.055 PMC4317728

[102] Liang K, Gao X, Gilmore JM, Florens L, Washburn MP, Smith E, et al. Characterization of human cyclin-dependent kinase 12 (CDK12) and CDK13 complexes in C-terminal domain phosphorylation, gene transcription, and RNA processing. Mol Cell Biol. 2015;35(6):928–38. 25561469 10.1128/MCB.01426-14 PMC4333096

[103] Anders L, Guenther MG, Qi J, Fan ZP, Marineau JJ, Rahl PB, et al. Genome-wide localization of small molecules. Nat Biotechnol. 2014;32(1):92–6. 24336317 10.1038/nbt.2776 PMC4189815

[104] Delmore JE, Issa GC, Lemieux ME, Rahl PB, Shi J, Jacobs HM, et al. BET bromodomain inhibition as a therapeutic strategy to target c-Myc. Cell. 2011;146(6):904–17. 21889194 10.1016/j.cell.2011.08.017 PMC3187920

[105] Filippakopoulos P, Qi J, Picaud S, Shen Y, Smith WB, Fedorov O, et al. Selective inhibition of BET bromodomains. Nature. 2010;468(7327):1067–73. 20871596 10.1038/nature09504 PMC3010259

[106] Ormsbee Golden BD, Gonzalez DV, Yochum GS, Coulter DW, Rizzino A. SOX2 represses c-MYC transcription by altering the co-activator landscape of the c-MYC super-enhancer and promoter regions. J Biol Chem. 2024;300(9):107642. 39122009 10.1016/j.jbc.2024.107642 PMC11408076

[107] Jaiswal SK, Fedkenheuer K, Khamar R, Tan H, Gotea V, Raj S, et al. The Megacomplex protects ER-alpha from degradation by Fulvestrant in epithelial ovarian cancer. Cancer Lett. 2025;608:217129. 39048045 10.1016/j.canlet.2024.217129 PMC12276986

[108] Song P, Han R, Yang F. Super enhancer lncRNAs: a novel hallmark in cancer. Cell Communication Signaling: CCS. 2024;22(1):207. 38566153 10.1186/s12964-024-01599-6 PMC10986047

[109] Suzuki HI, Young RA, Sharp PA. Super-enhancer-mediated RNA Processing revealed by Integrative MicroRNA Network Analysis. Cell. 2017;168(6):1000–e1415. 28283057 10.1016/j.cell.2017.02.015 PMC5350633

[110] Tolani B, Gopalakrishnan R, Punj V, Matta H, Chaudhary PM. Targeting Myc in KSHV-associated primary effusion lymphoma with BET bromodomain inhibitors. Oncogene. 2014;33(22):2928–37. 23792448 10.1038/onc.2013.242 PMC4892892

[111] Ceribelli M, Hou ZE, Kelly PN, Huang DW, Wright G, Ganapathi K, et al. A druggable TCF4- and BRD4-Dependent Transcriptional Network sustains malignancy in Blastic Plasmacytoid dendritic cell neoplasm. Cancer Cell. 2016;30(5):764–78. 27846392 10.1016/j.ccell.2016.10.002 PMC5175469

[112] Bhagwat AS, Roe JS, Mok BYL, Hohmann AF, Shi J, Vakoc CR. BET bromodomain inhibition releases the Mediator Complex from Select cis-Regulatory Elements. Cell Rep. 2016;15(3):519–30. 27068464 10.1016/j.celrep.2016.03.054 PMC4838499

[113] Yokoyama Y, Zhu H, Lee JH, Kossenkov AV, Wu SY, Wickramasinghe JM, et al. BET inhibitors suppress ALDH Activity by Targeting ALDH1A1 Super-enhancer in Ovarian Cancer. Cancer Res. 2016;76(21):6320–30. 27803105 10.1158/0008-5472.CAN-16-0854 PMC5117661

[114] Gilan O, Lam EY, Becher I, Lugo D, Cannizzaro E, Joberty G, et al. Functional interdependence of BRD4 and DOT1L in MLL leukemia. Nat Struct Mol Biol. 2016;23(7):673–81. 27294782 10.1038/nsmb.3249

[115] Coppé JP, Desprez PY, Krtolica A, Campisi J. The senescence-associated secretory phenotype: the dark side of tumor suppression. Annu Rev Pathol. 2010;5:99–118. 20078217 10.1146/annurev-pathol-121808-102144 PMC4166495

[116] Tasdemir N, Banito A, Roe JS, Alonso-Curbelo D, Camiolo M, Tschaharganeh DF, et al. BRD4 connects enhancer remodeling to Senescence Immune Surveillance. Cancer Discov. 2016;6(6):612–29. 27099234 10.1158/2159-8290.CD-16-0217 PMC4893996

[117] Arner E, Daub CO, Vitting-Seerup K, Andersson R, Lilje B, Drabløs F, et al. Transcribed enhancers lead waves of coordinated transcription in transitioning mammalian cells. Volume 347. New York, NY: Science; 2015. pp. 1010–4. 6225. 10.1126/science.1259418 PMC4681433 25678556

[118] Core LJ, Martins AL, Danko CG, Waters CT, Siepel A, Lis JT. Analysis of nascent RNA identifies a unified architecture of initiation regions at mammalian promoters and enhancers. Nat Genet. 2014;46(12):1311–20. 25383968 10.1038/ng.3142 PMC4254663

[119] Zhang T, Kwiatkowski N, Olson CM, Dixon-Clarke SE, Abraham BJ, Greifenberg AK, et al. Covalent targeting of remote cysteine residues to develop CDK12 and CDK13 inhibitors. Nat Chem Biol. 2016;12(10):876–84. 27571479 10.1038/nchembio.2166 PMC5033074

[120] Pelish HE, Liau BB, Nitulescu II, Tangpeerachaikul A, Poss ZC, Da Silva DH, et al. Mediator kinase inhibition further activates super-enhancer-associated genes in AML. Nature. 2015;526(7572):273–6. 26416749 10.1038/nature14904 PMC4641525

[121] Clarke PA, Ortiz-Ruiz MJ, TePoele R, Adeniji-Popoola O, Box G, Court W et al. Assessing the mechanism and therapeutic potential of modulators of the human mediator complex-associated protein kinases. eLife. 2016;5. 10.7554/eLife.20722 PMC5224920 27935476

[122] Kennedy AL, Vallurupalli M, Chen L, Crompton B, Cowley G, Vazquez F, et al. Functional, chemical genomic, and super-enhancer screening identify sensitivity to cyclin D1/CDK4 pathway inhibition in ewing sarcoma. Oncotarget. 2015;6(30):30178–93. 26337082 10.18632/oncotarget.4903 PMC4745789

[123] Mohamed AA, Xavier CP, Sukumar G, Tan SH, Ravindranath L, Seraj N, et al. Identification of a small molecule that selectively inhibits ERG-Positive Cancer cell growth. Cancer Res. 2018;78(13):3659–71. 29712692 10.1158/0008-5472.CAN-17-2949

[124] Winters B, Brown L, Coleman I, Nguyen H, Minas TZ, Kollath L, et al. Inhibition of ERG activity in patient-derived prostate Cancer xenografts by YK-4-279. Anticancer Res. 2017;37(7):3385–96. 28668826 10.21873/anticanres.11705

[125] Wang W, Tang YA, Xiao Q, Lee WC, Cheng B, Niu Z, et al. Stromal induction of BRD4 phosphorylation results in chromatin remodeling and BET inhibitor resistance in Colorectal Cancer. Nat Commun. 2021;12(1):4441. 34290255 10.1038/s41467-021-24687-4 PMC8295257

[126] Guo J, Liu Y, Lv J, Zou B, Chen Z, Li K et al. BCL6 confers KRAS-mutant non-small-cell lung cancer resistance to BET inhibitors. J Clin Investig. 2021;131(1). 10.1172/JCI133090 PMC7773368 33393503

[127] Ma Y, Wang L, Neitzel LR, Loganathan SN, Tang N, Qin L, et al. The MAPK pathway regulates intrinsic resistance to BET inhibitors in Colorectal Cancer. Clin cancer Research: Official J Am Association Cancer Res. 2017;23(8):2027–37. 10.1158/1078-0432.CCR-16-0453 PMC5368030 27678457

[128] Settleman J. Cancer: bet on drug resistance. Nature. 2016;529(7586):289–90. 26735017 10.1038/nature16863

[129] Wang L, Xu M, Kao CY, Tsai SY, Tsai MJ. Small molecule JQ1 promotes prostate cancer invasion via BET-independent inactivation of FOXA1. J Clin Investig. 2020;130(4):1782–92. 31874106 10.1172/JCI126327 PMC7108891

[130] Janouskova H, El Tekle G, Bellini E, Udeshi ND, Rinaldi A, Ulbricht A, et al. Opposing effects of cancer-type-specific SPOP mutants on BET protein degradation and sensitivity to BET inhibitors. Nat Med. 2017;23(9):1046–54. 28805821 10.1038/nm.4372 PMC5592092

[131] Dai X, Wang Z, Wei W. SPOP-mediated degradation of BRD4 dictates cellular sensitivity to BET inhibitors. Cell Cycle (Georgetown Tex). 2017;16(24):2326–9. 29108467 10.1080/15384101.2017.1388973 PMC5788415

[132] Dai X, Gan W, Li X, Wang S, Zhang W, Huang L, et al. Prostate cancer-associated SPOP mutations confer resistance to BET inhibitors through stabilization of BRD4. Nat Med. 2017;23(9):1063–71. 28805820 10.1038/nm.4378 PMC5625299

[133] Bradley CA. Prostate cancer: BET inhibitors - SPOP right there! Nat Rev Cancer. 2017;17(10):574–5. 28883513 10.1038/nrc.2017.80

[134] Zhang P, Wang D, Zhao Y, Ren S, Gao K, Ye Z, et al. Intrinsic BET inhibitor resistance in SPOP-mutated prostate cancer is mediated by BET protein stabilization and AKT-mTORC1 activation. Nat Med. 2017;23(9):1055–62. 28805822 10.1038/nm.4379 PMC5653288

[135] Borbely G, Haldosen LA, Dahlman-Wright K, Zhao C. Induction of USP17 by combining BET and HDAC inhibitors in breast cancer cells. Oncotarget. 2015;6(32):33623–35. 26378038 10.18632/oncotarget.5601 PMC4741790

[136] Fiskus W, Sharma S, Qi J, Valenta JA, Schaub LJ, Shah B, et al. Highly active combination of BRD4 antagonist and histone deacetylase inhibitor against human acute myelogenous leukemia cells. Mol Cancer Ther. 2014;13(5):1142–54. 24435446 10.1158/1535-7163.MCT-13-0770

[137] Latif AL, Newcombe A, Li S, Gilroy K, Robertson NA, Lei X, et al. BRD4-mediated repression of p53 is a target for combination therapy in AML. Nat Commun. 2021;12(1):241. 33431824 10.1038/s41467-020-20378-8 PMC7801601

[138] Mazur PK, Herner A, Mello SS, Wirth M, Hausmann S, Sánchez-Rivera FJ, et al. Combined inhibition of BET family proteins and histone deacetylases as a potential epigenetics-based therapy for pancreatic ductal adenocarcinoma. Nat Med. 2015;21(10):1163–71. 26390243 10.1038/nm.3952 PMC4959788

[139] Shahbazi J, Liu PY, Atmadibrata B, Bradner JE, Marshall GM, Lock RB, et al. The bromodomain inhibitor JQ1 and the histone deacetylase inhibitor panobinostat synergistically reduce N-Myc expression and induce Anticancer effects. Clin cancer Research: Official J Am Association Cancer Res. 2016;22(10):2534–44. 10.1158/1078-0432.CCR-15-1666 26733615

[140] Zhao L, Okhovat JP, Hong EK, Kim YH, Wood GS. Preclinical studies support combined inhibition of BET Family Proteins and Histone Deacetylases as epigenetic therapy for cutaneous T-Cell lymphoma. Neoplasia (New York NY). 2019;21(1):82–92. 10.1016/j.neo.2018.11.006 PMC6280696 30529073

[141] Chen J, Nelson C, Wong M, Tee AE, Liu PY, La T, et al. Targeted therapy of TERT-Rearranged neuroblastoma with BET Bromodomain Inhibitor and proteasome inhibitor combination therapy. Clin cancer Research: Official J Am Association Cancer Res. 2021;27(5):1438–51. 10.1158/1078-0432.CCR-20-3044 33310889

[142] Lam LT, Lin X, Faivre EJ, Yang Z, Huang X, Wilcox DM, et al. Vulnerability of small-cell lung Cancer to apoptosis Induced by the combination of BET Bromodomain Proteins and BCL2 inhibitors. Mol Cancer Ther. 2017;16(8):1511–20. 28468776 10.1158/1535-7163.MCT-16-0459

[143] Zhang W, Ge H, Jiang Y, Huang R, Wu Y, Wang D, et al. Combinational therapeutic targeting of BRD4 and CDK7 synergistically induces anticancer effects in head and neck squamous cell carcinoma. Cancer Lett. 2020;469:510–23. 31765738 10.1016/j.canlet.2019.11.027

[144] Xia L, Liu JY, Zheng ZZ, Chen YJ, Ding JC, Hu YH, et al. BRD4 inhibition boosts the therapeutic effects of epidermal growth factor receptor-targeted chimeric antigen receptor T cells in glioblastoma. Mol Therapy: J Am Soc Gene Therapy. 2021;29(10):3011–26. 10.1016/j.ymthe.2021.05.019 PMC8531146 34058385

[145] Karakashev S, Zhu H, Yokoyama Y, Zhao B, Fatkhutdinov N, Kossenkov AV, et al. BET bromodomain inhibition synergizes with PARP inhibitor in epithelial ovarian Cancer. Cell Rep. 2017;21(12):3398–405. 29262321 10.1016/j.celrep.2017.11.095 PMC5745042

[146] Yang L, Zhang Y, Shan W, Hu Z, Yuan J, Pi J et al. Repression of BET activity sensitizes homologous recombination-proficient cancers to PARP inhibition. Sci Transl Med. 2017;9(400). 10.1126/scitranslmed.aal1645 PMC5705017 28747513

[147] Miller AL, Fehling SC, Garcia PL, Gamblin TL, Council LN, van Waardenburg R, et al. The BET inhibitor JQ1 attenuates double-strand break repair and sensitizes models of pancreatic ductal adenocarcinoma to PARP inhibitors. EBioMedicine. 2019;44:419–30. 31126889 10.1016/j.ebiom.2019.05.035 PMC6604668

[148] Huang SH, Cao R, Lin QW, Wu SQ, Gao LL, Sun Q, et al. Design, synthesis and mechanism studies of novel dual PARP1/BRD4 inhibitors against pancreatic cancer. Eur J Med Chem. 2022;230:114116. 35091172 10.1016/j.ejmech.2022.114116

[149] Feng B, Yu H, Dong X, Díaz-Holguín A, Antolin AA, Hu H. Combining Data-Driven and structure-based approaches in Designing Dual PARP1-BRD4 inhibitors for breast Cancer Treatment. J Chem Inf Model. 2024;64(19):7725–42. 39292752 10.1021/acs.jcim.4c01421 PMC11480993

[150] Zhang J, Yang C, Tang P, Chen J, Zhang D, Li Y, et al. Discovery of 4-Hydroxyquinazoline derivatives as small molecular BET/PARP1 inhibitors that induce defective homologous recombination and lead to synthetic lethality for triple-negative breast Cancer Therapy. J Med Chem. 2022;65(9):6803–25. 35442700 10.1021/acs.jmedchem.2c00135

[151] Békés M, Langley DR, Crews CM. PROTAC targeted protein degraders: the past is prologue. Nat Rev Drug Discovery. 2022;21(3):181–200. 35042991 10.1038/s41573-021-00371-6 PMC8765495

[152] Li H, Dong J, Cai M, Xu Z, Cheng XD, Qin JJ. Protein degradation technology: a strategic paradigm shift in drug discovery. J Hematol Oncol. 2021;14(1):138. 34488823 10.1186/s13045-021-01146-7 PMC8419833

[153] Ma Z, Zhang C, Bolinger AA, Zhou J. An updated patent review of BRD4 degraders. Expert Opin Ther Pat. 2024;34(10):929–51. 39219068 10.1080/13543776.2024.2400166 PMC11427152

[154] Hsia O, Hinterndorfer M, Cowan AD, Iso K, Ishida T, Sundaramoorthy R, et al. Targeted protein degradation via intramolecular bivalent glues. Nature. 2024;627(8002):204–11. 38383787 10.1038/s41586-024-07089-6 PMC10917667

[155] Bauer K, Hauswirth A, Gleixner KV, Greiner G, Thaler J, Bettelheim P, et al. BRD4 degraders may effectively counteract therapeutic resistance of leukemic stem cells in AML and ALL. Am J Hematol. 2024;99(9):1721–31. 38822666 10.1002/ajh.27385

[156] Qian H, Zhu M, Tan X, Zhang Y, Liu X, Yang L. Super-enhancers and the super-enhancer reader BRD4: tumorigenic factors and therapeutic targets. Cell Death Discovery. 2023;9(1):470. 38135679 10.1038/s41420-023-01775-6 PMC10746725

[157] Yu J, Yan Y, Li S, Xu Y, Parolia A, Rizvi S, et al. Progestogen-driven B7-H4 contributes to onco-fetal immune tolerance. Cell. 2024;187(17):4713–e3219. 38968937 10.1016/j.cell.2024.06.012 PMC11344674

[158] Jia D, Wang Q, Qi Y, Jiang Y, He J, Lin Y, et al. Microbial metabolite enhances immunotherapy efficacy by modulating T cell stemness in pan-cancer. Cell. 2024;187(7):1651–e6521. 38490195 10.1016/j.cell.2024.02.022

[159] Sui Y, Wang T, Mei Y, Zhu Y, Jiang W, Shen J, et al. Targeting Super-enhancer-driven Transcriptional dependencies suppresses aberrant hedgehog pathway activation and overcomes smoothened inhibitor resistance. Cancer Res. 2024;84(16):2690–706. 38775809 10.1158/0008-5472.CAN-23-3306

